# Staph wars: the antibiotic pipeline strikes back

**DOI:** 10.1099/mic.0.001387

**Published:** 2023-09-01

**Authors:** Edward J.A. Douglas, Maisem Laabei

**Affiliations:** ^1^​ Department of Life Sciences, University of Bath, Bath BA2 7AY, UK

**Keywords:** antimicrobial resistance, antibiotic therapy, *Staphylococcus aureus*

## Abstract

Antibiotic chemotherapy is widely regarded as one of the most significant medical advancements in history. However, the continued misuse of antibiotics has contributed to the rapid rise of antimicrobial resistance (AMR) globally. *Staphylococcus aureus *, a major human pathogen, has become synonymous with multidrug resistance and is a leading antimicrobial-resistant pathogen causing significant morbidity and mortality worldwide. This review focuses on (1) the targets of current anti-staphylococcal antibiotics and the specific mechanisms that confirm resistance; (2) an in-depth analysis of recently licensed antibiotics approved for the treatment of *S. aureus* infections; and (3) an examination of the pre-clinical pipeline of anti-staphylococcal compounds. In addition, we examine the molecular mechanism of action of novel antimicrobials and derivatives of existing classes of antibiotics, collate data on the emergence of resistance to new compounds and provide an overview of key data from clinical trials evaluating anti-staphylococcal compounds. We present several successful cases in the development of alternative forms of existing antibiotics that have activity against multidrug-resistant *S. aureus *. Pre-clinical antimicrobials show promise, but more focus and funding are required to develop novel classes of compounds that can curtail the spread of and sustainably control antimicrobial-resistant *S. aureus* infections.

## Introduction

The World Health Organization (WHO) has identified antimicrobial resistance (AMR) as one of the gravest threats to global public health due to the significant morbidity, mortality and economic costs associated with multidrug-resistant (MDR) infections [1]. For example, in the USA, over 2.8 million people suffer from antibiotic-resistant infections annually, resulting in more than 35 000 deaths [2]. Furthermore, disease caused by antibiotic-resistant pathogens results in extra healthcare costs of approximately US $20 billion to US hospitals. Future predictions estimate that if left unchecked, AMR could lead to 10 million deaths per year and negatively impact global GDP to the tune of US $100 trillion [3].

*Staphylococcus aureus* is a major human pathogen and leading cause of bacteraemia and infective endocarditis, as well as bone, skin and soft tissue, pleuropulmonary and implant-related infections [4]. The range and severity of diseases caused by *S. aureus* are in part determined by the myriad of virulence factors, including toxins, adhesins, immune evasins, proteases and other tissue-degrading enzymes. Combined with the impressive ability to gain resistance to multiple classes of antibiotics, these attributes culminate in a pathogen that can be incredibly difficult to treat. At present, there is no available vaccine to prevent *S. aureus* infection. All previous vaccination attempts have failed at clinical trials, particularly those designed to generate high titres of opsonic antibodies directed against staphylococcal surface antigens [5, 6]. The complex host–pathogen interaction has led to several hypotheses for the disappointing results observed in human trials, which have been extensively reviewed [5–7]. Importantly, lack of effective vaccines significantly enhances the clinical use of antibiotics for treatment of *S. aureus* infections, increasing the selective pressure for resistance. Worryingly, recent reports have estimated that *S. aureus* ranks second in global AMR burden, with 100 000 deaths attributed to, and 1 million deaths associated with, resistant infections caused by this bacterium [8].

Current anti-staphylococcal treatment options rely on antibiotics with well-defined bacterial targets that are described in detail below along with the mechanisms that *S. aureus* uses to resist their activity. The treatment regimen to combat *S. aureus* depends on several factors, including disease severity and site of infection, antibiogram of the causative isolate and patient antibiotic allergies, and has been recently reviewed [4, 9].

*S. aureus* AMR mechanisms fall into four main categories: (1) limiting drug uptake, (2) modifications of the target site, (3) drug inactivation and (4) active drug efflux. These resistance mechanisms can either be *intrinsic,* the resistance genes are chromosomally located and expressed or induced in all species, or they can be *acquired*. The most common examples of intrinsic resistance in *S. aureus* include limiting drug uptake, typically through cell wall modifications, or drug efflux. Acquired resistance arises either from the gain of genetic material through horizontal gene transfer or chromosomal mutations. Genotypic evidence indicates that clinically derived *S. aureus* isolates utilize combinations of these resistance mechanisms that have been employed to withstand the activity of all known classes of antibiotics [10, 11]. *S. aureus* can be considered MDR by virtue of harbouring the *mecA* gene or when resistant to ≥1 antibiotic agent in ≥3 antibiotic categories. The increase in the antibiotic resistance coverage of *S. aureus* has resulted in a new antimicrobial category called extensively drug-resistant (XDR), which can be classified as an isolate that is resistant to ≥1 antibiotic agent in all but ≤2 antibiotic categories [12]. While still relatively rare, XDR *S. aureus* isolates are increasingly being reported and represent a significant health concern [13, 14]. Pan-drug resistance (PDR) has currently not been reported in *S. aureus,* potentially explained by the fitness cost associated with such resistance. This is in stark contrast to the Gram-negative organisms *Klebsiella pneumoniae *, *Acinetobacter baumanii* and *Pseudomonas aeruginosa,* whereby PDR is increasingly being documented worldwide and is associated with high mortality [15, 16].

Compounding the problem surrounding *S. aureus* antibiotic resistance, is the lack of innovation in antibiotic development, where few novel antibiotic classes have been introduced successfully into the clinic since the turn of the century. Indeed, the last truly novel Gram-positive antibiotics introduced were daptomycin (DAP) and linezolid (LZD) in the early 2000s [17]. Accordingly, this review aims to provide a background to recently licensed anti-staphylococcal antibiotics, while providing an assessment of the current pipeline and promising prospects. We aim to provide a critical analysis of the antibiotics that are currently in the mid to late stage of the clinical development process, indicating their mechanism of action, where applicable how they differ from previous structures, whether resistance has been observed, and an overview of the current clinical trial data. Importantly, while numerous antibiotics in development have anti-staphylococcal activity, we are focused on those that are specifically being developed to combat *S. aureus *.

## Current anti-staphylococcal antibiotics: targets and resistance mechanisms

### The cell wall

β-lactams, such as penicillin (PEN), bear a structural resemblance to the d-Ala-d-Ala-terminating pentapeptide of lipid II, which is the natural substrate for transpeptidase penicillin-binding proteins (PBPs). Thus β-lactams interrupt cell wall formation by covalently binding to PBPs and preventing the transpeptidase reaction from occurring, resulting in cell death [18]. The resistance of *S. aureus* to this class of antibiotic has garnered this bacterium much notoriety and it has become a global health burden. Resistance first emerged against PEN in the 1940s, a few years after it was introduced into the clinic [19]. Resistant strains acquired a plasmid encoding a penicillinase called *blaZ*, which was able to hydrolyse the β-lactam ring, rendering it unable to bind to PBPs [20]. PEN resistance spread rapidly; by the 1960s more than 90 % of strains produced this enzyme regardless of clinical isolation source [21, 22]. As a response, a semi-synthetic PEN, containing a dimethoxyphenyl group called methicillin (MET), insensitive to penicillinase activity, was introduced. *S. aureus* resistance to this modified β-lactam emerged rapidly and strains were termed methicillin-resistant *S. aureus* (MRSA) [23]. A low-affinity PBP called PBP2A/PBP2′ encoded by the *mecA* gene harboured on a mobile genetic element, the staphylococcal chromosomal cassette *mec* (SCC*mec*), was discovered to confer resistance to MET and certain cephalosporins [24–26].

Glycopeptides similarly inhibit cell wall synthesis; however, these antibiotics bind to the d-Ala-d-Ala residue of lipid II, which results in a cessation of the transpeptidase reaction through substrate sequestration but also a reduction in transglycosylase activity through additional steric hindrance [27, 28]. Glycopeptides can also be called lipoglycopeptides when a lipophilic side chain is present in the structure, as is the case with teicoplanin and telavancin [29]. The unprecedented spread of MRSA globally led clinics to rely on glycopeptides such as vancomycin (VAN), which were previously considered ‘last resort’ antimicrobials. This increased selective pressure eventually led to the emergence of hetero-vancomycin intermediate *S. aureus* (hVISA), which served as a precursor to vancomycin intermediate *S. aureus* (VISA) with minimal inhibitory concentrations (MICs) of ≥4 µg ml^−1^ [30, 31]. The emergence of VISA is attributed to chromosomal mutations in cell wall stress stimulon genes such as *walRK*, *yycH*, *vraRS*, *graRS*, *rpoB* and *tcaA* [32]. These mutations serve to decrease the level of crosslinking present within the cell wall, resulting in an increase in the number of free d-Ala-d-Ala residues. Thus, VAN is sequestered within the cell wall instead of reaching its lipid II target at the cell membrane. This mechanism of resistance has been referred to as ‘drug capture ‘or ‘clogging’ [32]. It was feared that VISA could in turn lead to the emergence of vancomycin-resistant *S. aureus* (VRSA). Unfortunately, this has since come to fruition due to the acquisition of the *vanA* gene, mobilized on the *Tn*1536 transposable element, from *Enterococcus faecium* [33]. The expression of this gene alters the early stages of peptidoglycan synthesis, replacing the terminal d-Ala on lipid II with d-lactate. This change causes a >1000-fold decrease in VAN binding affinity [33, 34]. So far, VRSA has not spread in the same way as penicillin-resistant *S. aureus* or MRSA. Instead, only isolated reports have been described and these strains do not seem to have established themselves in healthcare facilities [35]. The reasoning for this is threefold. Firstly, PBP2A struggles to utilize d-Ala-d-Lac as a substrate for peptidoglycan transpeptidation. Secondly, VRSA strains exhibit an extremely long lag phase before growth in inhibitory concentrations of VAN is initiated. Finally, the plasmids harbouring *vanA* were found to be unstable in *S. aureus* [11].

### The folate pathway

TMP–SMX is a synergistic combination of two antibiotics that work by inhibiting the formation of folic acid, an essential nutrient for nucleic acid synthesis [36]. Sulfamethoxazole (SMX) is an antibiotic that is structurally analogous to para-aminobenzoic acid (PABA). This enables it to bind to the dihydropteroate synthase enzyme (DHPS) and prevent the conversion of PABA to dihydropteroate (DHP) [37]. SMX resistance is achieved through chromosomal overproduction of PABA [38] or through mutational changes in the *folP* gene, which encodes DHPS, which presumably reduces the binding affinity of SMX [39, 40]. Trimethoprim (TMP) acts later in the folic acid biosynthesis pathway, instead inhibiting the dihydrofolate reductase (DHFR) enzyme, thereby preventing the formation of tetrahydrofolate (THF) from dihydrofolate (DHF) [41]. TMP resistance is achieved through a single point mutation F98Y in the chromosomal *dfrB* gene, which codes for the DHFR enzyme. This confers intermediate resistance, with MICs ≤256 µg ml^−1^ [42]. For high-level resistance, MICs of ≥512 µg ml^−1^, *S. aureus* must acquire alternative naturally occurring resistant *dfr* genes such as *dfrA*, *dfrG* and *dfrK* from *Bacteroides *, *Clostridium* and *Neisseria* species, which can be housed on plasmids or transposable elements [43–45]. A further novel diaminopyrimidine resistance determinant, designated *dfrL,* was found recently in a livestock-associated MRSA strain of clonal complex 398. This variant conferred a TMP MIC of 1024 µg ml^−1^. The ancestry of the *dfrL* is unclear, but the authors suggested that it is of recent staphylococcal origin [46].

### The cell membrane

The mechanism of action of the lipopeptide DAP is not fully understood. DAP requires Ca^2+^ ions for antimicrobial activity facilitating the formation of an amphiphilic complex, similar to cationic antimicrobial peptides (cAMPs). As cAMPs target the negatively charged phospholipids of the Gram-positive bacterial membrane, researchers initially postulated that DAP would have a similar mechanism of action [47]. Indeed, numerous membrane perturbation effects have been described for DAP, including altered membrane curvature, membrane depolarization and leakage [48]. However, kill kinetic analysis has suggested this to be the result of rather than the cause of death [49]. More recent reports describe a role for DAP in cell wall inhibition. It was shown that DAP interacts with undecaprenyl-coupled cell wall precursors and phosphatidylglycerol to form a tripartite complex resulting in delocalization of peptidoglycan machinery and significant membrane rearrangement [50]. Thus, DAP could equally be categorized as an antibiotic that targets the cell wall. Resistance to DAP is typically achieved through point mutations in MprF, which catalyses the synthesis and translocation of the positively charged phospholipid lysyl-phosphatidylglycerol into the outer leaflet of the membrane [51]. It is unclear how these mutations impact on DAP resistance. Initial observations suggested that single-nucleotide polymorphisms (SNPs) were gain of function, causing an increase in the membrane levels of lysyl-phosphatidylglycerol that would cause electrostatic repulsion of DAP [52]. In agreement with this, a change in surface charge can often be detected for strains harbouring *mprF* mutations. However, this is not always the case and some *mprF* mutations, although causing an increase in DAP MIC, are not associated with an increase in lysyl-phosphatidylglycerol levels [53, 54]. More recently, crosstalk between MprF and cell wall biosynthesis machinery was observed where mutations in *mprF* stimulated the expression of the *vraRS* two-component system, resulting in a thicker cell wall [55, 56]. Accordingly, MprF-mediated DAP resistance appears to be dual functional, altering the architecture of both the cell wall and the cell membrane. Resistance to DAP can also occur through an increase in d-alanylation of teichoic acids, a process that decorates the teichoic acid polymers with positive charges, which serves to repel DAP [57, 58]. Other genes involved in resistance to DAP include the cardiolipin synthase *cls,* the phospholipid synthase *pgsA* and *rpoB* which codes for the β-subunit of the DNA-dependant RNA polymerase [59].

### Nucleic acid biosynthesis

Fluoroquinolones, such as levofloxacin (LVX), comprise the first entirely synthetic antibiotic class that work by targeting two enzymes involved in DNA replication. The first enzyme, DNA gyrase, is formed of a heterotetramer of GyrA and GyrB, and catalyses the ATP-dependent negative supercoiling of DNA [60]. The second enzyme, DNA topoisomerase IV, consists of a heterotetramer of GrlA and GrlB (also referred to as ParC and ParE), which promotes chromosome decatenation following replication [60]. Together these enzymes work to relieve the enormous torsional stress and topological constraints present during DNA replication. Inhibition of these enzymes results in DNA shearing preventing replication [60, 61]. Resistance to fluoroquinolones in *S. aureus* occurs through two main mechanisms. The first is alteration of the target enzymes caused by single or multiple point mutations of either gyrase or topoisomerase, which reduce the binding affinity of the fluoroquinolone [62–64]. The second is increased efflux; overexpression of the NorA [65], NorB [66] and NorC [67] efflux pumps has been shown to cause a four- to eightfold increase in MIC against various fluoroquinolones. These efflux pumps belong to the major facilitator superfamily (MFS) of transporters that utilize the proton gradient across the bacterial membrane to expel substrates. NorA confers resistance against hydrophilic fluroquinolones such as ciprofloxacin (CIP). The NorB and NorC efflux are less stringent and can expel both hydrophilic and hydrophobic fluoroquinolones [65–68]. A number of other *S. aureus* MFS transporters have also been shown to contribute to fluoroquinolone resistance but are less well studied than the Nor efflux pumps. Hyper-production of MdeA and QacB reduces the susceptibility towards CIP and norfloxacin (NOR) [69, 70]. SdrM and LmrS also reduce susceptibility towards NOR and gatifloxacin, respectively [71, 72]. MepA, a member of the multiple antibiotic and toxin extrusion (MATE) family of secondary transporters, also confers elevated MICs against CIP, NOR, moxifloxacin (MOX) and sparfloxacin [73]. Highly resistant strains will likely employ both of these mechanisms, often possessing multiple gyrase and topoisomerase IV mutations as well as being hyper-producers of one or more efflux pumps.

Ansamycins such as rifampicin (RIF) inhibit RNA synthesis by targeting the DNA-dependent RNA polymerase, binding within the DNA/RNA channel of the RNA polymerase β-subunit in close vicinity to the enzyme active site [74]. Rather than preventing RNA polymerase from binding to the promoter and initiating transcription, RIF inhibits the formation of the first ribonucleotide phosphodiester bond, halting mRNA production [75]. RIF resistance occurs through target site modification; mutations of the *rpoB* gene occur in a hotspot region termed the rifampicin resistance-determining region. By far the most encountered *rpoB* resistant mutations are H481Y and L466S [76, 77]. Highly resistant strains may harbour both these mutations along with others such as A477D and S486L [76–78]. Some *rpoB* mutations are associated with increased cell wall thickness, can promote the conversion of strains from hVISA to VISA and small colony variant (SCV) formation, significantly influencing the chronicity of *S. aureus* infections [79, 80].

### Protein synthesis

**Fig. 1.**
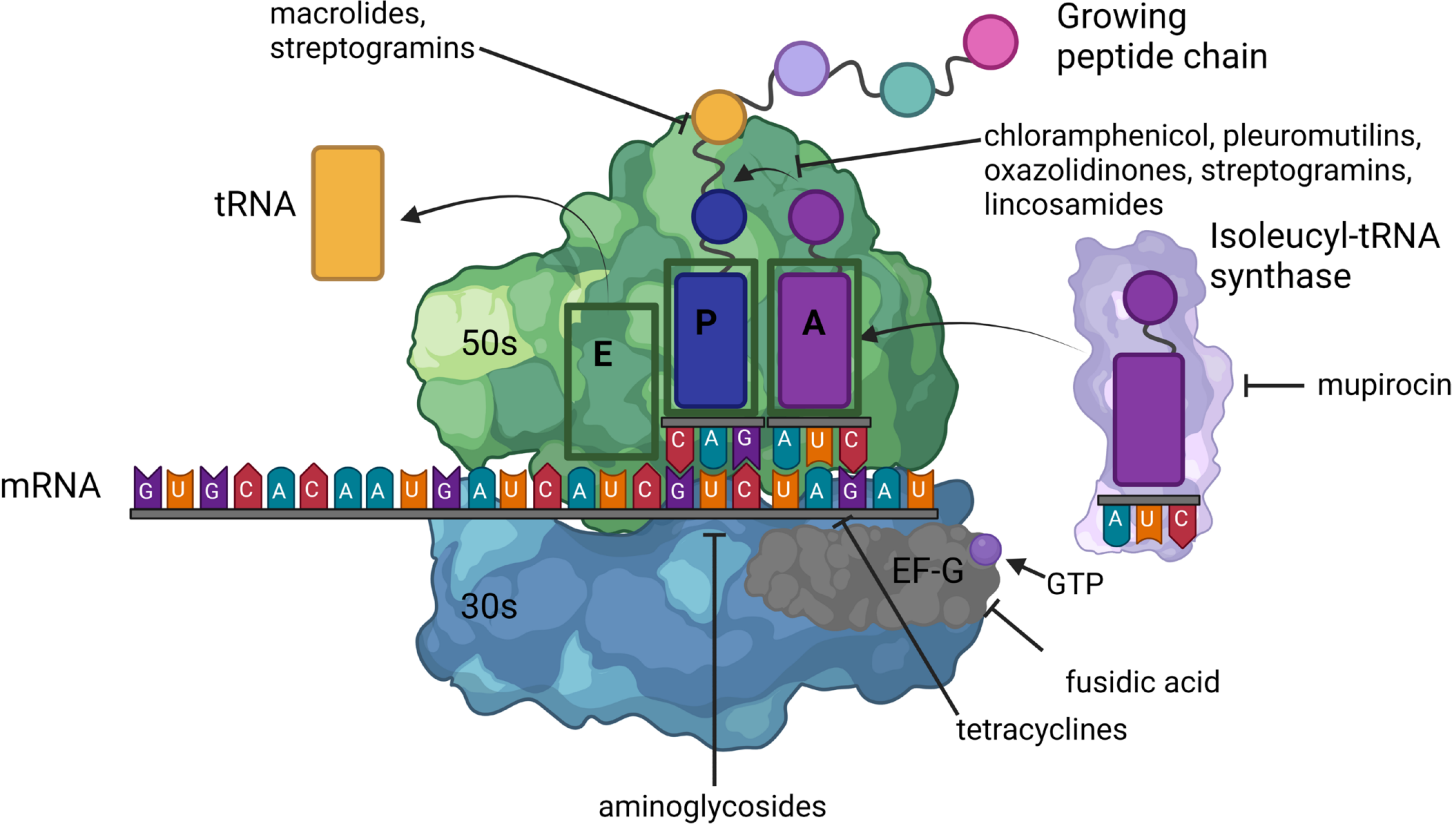
Ribosomal structure and antibiotics that interfere with protein synthesis**.** Ribosomes are central to the process of protein synthesis. In *S. aureus *, the ribosome is a 70S ribonucleoprotein complex consisting of a large 50S and small 30S subunit. The 50S subunit is formed from 23S and 5S ribosomal RNA (rRNA) and is responsible for initial amino acyl-tRNA binding, peptidyl transfer and regulation of the elongation factor. Three amino acyl-tRNA-binding sites are present within the 50S subunit. The 30S subunit is made from 16s rRNA and through binding to mRNA, initiates the translation process. Protein synthesis can be divided into four steps: initiation, elongation, termination and recycling. Binding of the 30S subunit to mRNA triggers the formation of the 70S complex and recruitment of the initiator tRNA (typically fMet-tRNA). Elongation follows and involves the addition of amino acids to a growing chain in a stepwise manner through the acceptor (A), peptidyl (P) and exit (E) amino acyl-tRNA-binding sites. Antibiotics that target the 50S subunit either block the polypeptide exit channel (macrolides and streptogramins) or disrupt amino acyl-tRNA binding at the peptidyl transferase centre (PTC) (lincosamides, streptogramins, phenicols, pleuromutilins and oxazolidinones). Tetracyclines bind to the 30S subunit, close to the decoding centre, and cause amino acyl-tRNA to dissociate from the A site. Aminoglycosides cause misreading of mRNA. Mupirocin inhibits the isoleucyl-tRNA synthase. Fusidic acid inhibits elongation factor G (EF-G), preventing the ribosome from traversing along the mRNA [11, 83, 288].

Multiple antibiotic classes inhibit protein synthesis. Antibiotics can be categorized into those that inhibit the 30S subunit of the ribosome such as the aminoglycosides and tetracyclines, compounds that inhibit the 50S subunit of the ribosome such as oxazolidinones, macrolides, streptogramins, lincosamides and pleuromutilins, and drugs with distinct mechanisms of action independent of these subunits, such as mupirocin and fusidic acid (Fig. 1). Several resistance mechanisms that prevent protein synthesis inhibition, particularly those directed against the 50S subunit, have substantial overlap (Table 1).

**Table 1.** Summary of resistance mechanisms used to evade protein synthesis inhibitors

Mechanism	Determinant	Spectrum
*30S ribosomal inhibitors*		
Drug inactivation	Ant, Aph, Aac	A
Efflux	TetK, TetL	T
Ribosomal protection	TetO, TetM	T
*50S ribosomal inhibitors*		
rRNA methyltransferase	Erm	MLS_B_
	Cfr	PhLOPS_A_
	Vga	LS_A_
	Lsa	LS_A_P
ABC-F ribosomal protection	Sal	LS_A_P
	Msr	MS_B_
	OptrA	OPh
Efflux	Fex	Ph
Drug modification	VgB	S_B_
	Vat	S_A_
	Cat	Ph
Target site mutation:23srRNA, L3 and L4		O
Target site modification	RluC	O
	RlmN	O
*Ribosome-independent protein synthesis inhibitors*		
Target site mutation	FusA, FusE	F_A_
Target site modification	FusB, FusC	F_A_
Target bypass	MupA	Mu

A, aminoglycosides; F_A_, fusidic acid; L, lincosamide; M, macrolide; Mu, mupirocin; O, oxazolidinones; P, pleuromutilins; Ph, chloramphenicol; S_A_, streptogramin A; S_B_, streptogramin B; T, tetracycline.

Aminoglycosides are the only ribosome-targeting antibiotics that are bactericidal. This is due to a distinctive mechanism of action that causes the misreading of codons. Consequently, the translational error rate is inflated from <1 in 1000 to around 1 in 100, so that almost every protein has multiple incorrect amino acids [81]. The bactericidal effect associated with aminoglycosides is thought to stem from the result of faulty membrane proteins that cause membrane channels to form and the leakage of intracellular contents [82]. The binding to the negatively charged phosphate groups of the 16S rRNA A site within the 30S subunit is facilitated by the presence of several amino and guanido groups within the aminoglycoside structure, which confers polycationic properties at physiological pH [83]. *S. aureus* resistance to aminoglycosides relies on the acquisition of aminoglycoside-modifying enzymes (AMEs), *aac (6′)-Ie+aph (2*″), ant (4′)-Ia, *aph (3′)-IIIa* and *ant (6)-Ia* genes, which serve to inactivate the antibiotic [84–86].

**Fig. 2.**
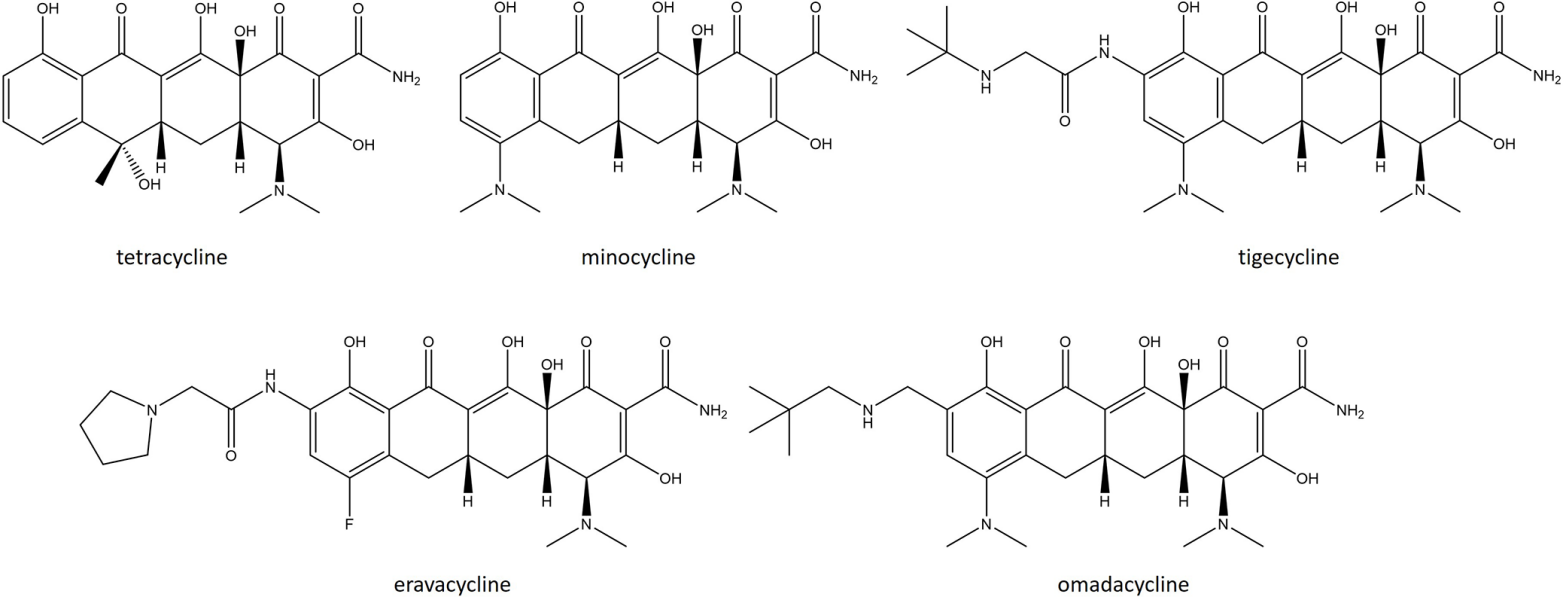
Structures of recently approved tetracyclines.

Tetracyclines prevent the binding of amino acyl tRNA through interactions with the 16S rRNA A site. Two main mechanisms are used by *S. aureus* to evade their killing. The first is active expulsion of the antibiotic through plasmid-mediated TetK and TetL MFS efflux pumps. TetK is typically housed on the small multicopy pT181 plasmid within the SCC*mecIII* of MRSA strains [87, 88]. A novel MFS efflux pump, SA09310, has also recently been shown to mediate tetracycline (TET) resistance; a Δ*sa09310* mutant displays a 64-fold and 8-fold reduction in MIC against TET and doxycycline (DOX) [89]. The second is through the expression of ribosomal protection proteins (RPPs), TetO and TetM, which bind to the ribosome and prevent the access of TET to their target. These proteins possess GTPase activity like elongation factor G (EF-G) but cannot function as an elongation factor and can either be chromosomally encoded or present on transposable elements such as Tn*916* and Tn*1545* [88, 90]. A third-generation semisynthetic TET called tigecycline (TGC) was introduced into the clinic in 2005. This newer derivative can be categorized separately to archetypal tetracyclines and referred to as a glycylcycline. It was designed by altering minocycline (MIN), specifically via the addition of a 9-tert-butyl-glycylamido side chain at the ninth position of the d-ring (Fig. 2) [91]. This side chain helps to overcome these two existing TET resistance mechanisms. The resulting molecule is substantially more potent than TET and MIN and active against strains harbouring tetracycline efflux pumps and RPPs [11]. However, TGC-resistant MRSA strains have since been reported and are caused by mutations in *mepR*, causing overexpression of MepA, and *rpsJ* [92].

Chloramphenicol (CHL) functions by binding reversibly to the L16 protein of the 50S ribosomal subunit. L16 interacts directly with the 23S rRNA in the 50S peptidyltransferase centre and thus CHL specifically disrupts peptide bond formation [11, 93]. In *S. aureus,* resistance occurs through several mechanisms. Acquisition of the chloramphenicol florfenicol resistance *cfr* gene encoding a ribosomal RNA methyltransferase that methylates an adenine residue at position 2503 in domain V of the 23S rRNA prevents CHL activity. Importantly, this gene confers an extended spectrum of resistance that includes oxazolidinones, phenicols, lincosamides, pleuromutilins and streptogramin A, commonly referred to as the PhLOPS_A_ group of antibiotics [94]. In addition, acquisition of the *cat* gene, encoding a CHL acetyltransferase that transfers an acetyl group from acetyl coenzyme A to CHL, results in drug inactivation. *S. aureus* may acquire the oxazolidinone phenicol resistance determinant *optrA,* conferring resistance to CHL and oxazolidinones. OptrA shares phylogenetic similarities with two ATP-binding site ABC-F proteins and is thought to displace the antibiotic from the target site. Lastly, resistance to CHL may occur through acquisition of *fexA*, which functions as an MFS transporter to expel CHL [95, 96].

Macrolides, lincosamides and streptogramins are often grouped together and referred to as the MLS group of antibiotics due to having a similar mechanism of action that involves targeting the 23S rRNA of the 50S ribosomal subunit and having overlapping resistance mechanisms [11]. Antibiotic binding at this site prevents the exit path of the growing peptide chain, which leads to a dissociation of the peptidyl-tRNA during the translocation reaction [97]. Resistance to MLS antibiotics is determined through the expression of either *erm* or *msr* genes. The *erm* genes (*ermA*, *ermB* and *ermC*) each encode adenyl-N-methyltransferase enzymes, which serve to alter the target site by specifically methylating a highly conserved adenine 2058 residue in domain V of the 23S rRNA. This triggers a conformational change in the ribosome, facilitating resistance to MLS antibiotics [98, 99]. The mechanism by which the *msr* genes *msrA* and *msrB* confer resistance is a contentious topic. *Msr* genes encode ATP-binding cassette (ABC) transporters, but it is unclear whether they function as dedicated efflux pumps or RPPs [100–102]. An additional mechanism of resistance against macrolides includes esterases encoded by *empC*, *ereA* and *ereB*, which hydrolyse the macrocylic lactone ring, promoting high-level erythromycin (ERY) resistance [100, 103]. Additional streptogramin/lincosamide resistance mechanisms include an alternative ABC transporter ribosomal protection protein called Sal [104]. Finally, virginiamycin B lyases (Vgb) can linearize streptogramin B molecules and virginiamycin acetyltransferases (Vat) O-acetylate the hydroxyl group at position O8 of streptogramin A molecules, leading to drug inactivation [105].

Oxazolidinones such as LZD bind to the 23S rRNA of the 50S ribosomal subunit close to the macrolide- and CHL-binding site but with a separate mechanism of action. These antibiotics interfere with the formation of the ribosomal–fMet–tRNA initiation complex. LZD is the first fully synthetic ribosomal-targeting antibiotic and, along with DAP, the only new antibiotic class to be introduced since 2000. Since its inception in the clinic, it has become invaluable in the treatment of complicated MRSA infections [106]. Along with resistance mediated by Cfr and OptrA, resistance to oxazolidinones can also be achieved through target site modification of the ribosome. In *S. aureus,* the most clinically relevant ribosomal mutation is G2576U, but U2500A and G2447U may also be encountered, although all three seem to have a direct impact on drug binding [107, 108]. Other oxazolidinone resistance mechanisms include upregulation of the RluC enzyme responsible for pseudouridylation of U2504, loss of RlmN methyltransferase activity and mutations in the ribosomal proteins L3 and L4 [109]. It is unclear whether L3/L4 mutations alone can cause LZD mutations, as they are typically present in strains already harbouring ribosomal mutations [108].

Pleuromutilins inhibit protein synthesis by binding close to the peptidyl transferase centre of the 50S ribosomal subunit. This interferes with the transfer of amino acyl-tRNAs from the A to the P site during peptidyl transfer, inhibiting peptide bond formation [110]. Along with Cfr, resistance to pleuromutilins is achieved through mutations in the *rplC* and *rplD* genes that encode the L3 and L4 ribosomal proteins. These proteins do not interact directly with pleuromutilins but can cause conformational changes in the peptidyl transferase centre, preventing correct positioning of the drug. A third cause of pleuromutilin resistance in *S. aureus* is through the *vga* family of genes that encode ABC-F transporters that lack transmembrane domains. Rather than contributing to efflux, it is thought that these proteins instead act as ribosomal protectors. These RPPs also confer resistance to streptogramin A and lincosamides [110, 111].

Fusidic acid (FA) is unusual in that it inhibits protein synthesis independent of ribosome binding, peptide bond or translocation inhibition, instead interfering with EF-G function [112]. EF-G functions as a GTPase that promotes the translocation of amino acyl-tRNA through the three ribosomal tRNA-binding sites, allowing deacylated tRNA to be expelled through the E site [112]. FA does not bind to free EF-G, instead it binds to an intermediary ribosome–EF-G complex, specifically at an inter domain pocket close to the GTPase active site. Thus, FA prevents EF-G dissociation from the ribosome and consequently the turnover of EF-G [113, 114]. There are two mechanisms by which *S. aureus* gains resistance to FA. The first is mutations of the gene encoding EF-G (*fusA*) or the gene encoding the ribosome protein L6 (*fusE*), which interacts with EF-G upon ribosome binding. Single mutations of the former are largely responsible for fusidic acid resistance in *S. aureus* [115, 116]. The second is horizontal gene acquisition of *fusB* and *fusC,* typically housed on SCC*mec* elements. These two genes encode proteins that bind to EF-G–fusidic acid complex that stimulates a conformational change within EF-G, allowing it to be released and for translation to continue [117, 118].

Mupirocin (MUP) also has a unique mechanism of action, inhibiting protein synthesis by targeting isoleucyl-tRNA synthase activity. This enzyme is responsible for the formation of a covalent linkage between the isoleucine amino acid and the respective tRNA for isoleucine tRNA^ile^ [119]. In the presence of mupirocin there is no tRNA carrying isoleucine available, thus protein synthesis halts at a codon for isoleucine. Low-level resistance to mupirocin is achieved through mutations in the isoleucyl-tRNA synthase that reduce the binding affinity of the drug [120]. Higher level resistance can be achieved through the gain of the plasmid housed determinant MupA. MupA encodes an insensitive isoleucyl-tRNA synthase, reminiscent of PBP2A with MET, which mupirocin is unable to bind [121, 122].

## Approved anti-staphylococcal antibiotics since 2010

Since 2010 there have been 15 antibiotics approved for clinical use that show excellent anti-staphylococcal activity (Table 2). Importantly, none were first-in-class antibiotics. Instead, these compounds were modifications of existing, well-established pharmacophores such as the cephalosporin, quinolone, oxazolidinone, tetracycline, pleuromutilin and lipoglycopeptide classes of antibiotic. Despite this seemingly impressive number of new antibiotics hitting the market, there is a marked disparity in their availability, with most only approved for use by US, EU, or UK medicinal governing bodies.

**Table 2.** Antibiotics launched since 2010. Antibiotics are ordered from oldest to newest and include trade name, company, approval year, drug class, route of administration, indications and countries (or regions) with approval

Antibiotic	Trade name	Company	Approval year	Drug class	Route of administration	Indications	Approval
Ceftaroline	Teflaro/Zinfloro	Allergen	2010	Cephalosporin	IV	ABSSSIs, CABP	FDA, EMA, MHRA
Dalbavancin	Dalvance	Allergen	2014	Lipoglycopeptide	IV	ABSSSIs	FDA, EMA, MHRA
Oritavancin	Orbactiv/Kirmyrsa	Melinta	2014	Lipoglycopeptide	IV	ABSSSIs	FDA. EMA, MHRA
Tedizolid	Sivextro	Merck	2014	Oxazolidinone	IV, po	ABSSSIs	FDA, EMA, MHRA
Finafloxacin	Xtoro	MerLion Pharmaceuticals	2014	Quinolone	Drop suspension	Titis externa	FDA, Health Canada
Nemonoxacin	Taigexyn	TaiGen Biotechnology	2016	Quinolone	IV, po	ABSSSIs, CABP	Taiwan ROC, Russian Federation, CFDA
Zabofloxacin	Zabolante	Dong Wha Pharmaceutical	2016	Quinolone	po	COPD	Republic of Korea, CFDA, Middle East, Africa
Delafloxacin	Baxdela/Quofenix	Melinta	2017	Quinolone	IV, po	ABSSSIs, CABP	FDA, EMA, MHRA
Ozenoxacin	Ozanex /Ozewid/Xepi	Medimetriks Pharmaceuticals	2017	Quinolone	Topical	Impetigo	EMA, PMDA, FDA, MHRA
Eravacycline	Xereva	Tetraphase	2018	Tetracycline	IV	cIAIs	FDA, EMA, MHRA
Omadacycline	Nurzyra	Paratek	2018	Tetracycline	IV, po	ABSSSIs, CABP	FDA
Lefamulin	Xenleta	Nabriva	2019	Pleuromutilin	IV, po	CABP	FDA, EMA
Lascufloxacin	Lasvic	Kyorin Pharmaceuticals	2019	Quinolone	IV, po	CABP	PMDA
Levonadifloxacin	Emrok	Wockhardt	2020	Quinolone	IV, po	ABSSSIs	DCGI
Contezolid	Youxitai	MicuRx	2021	Oxazolidinone	IV, po	cSSSIs	CFDA

ABSSSI, acute bacterial skin and skin structure infections; CABP, community acquired bacterial pneumonia; CFDA, China Food and Drug Administration ; cIAI, complicated intra-abdominal infection; COPD, chronic obstructive pulmonary disease; cSSSI, complicated bacterial skin and skin structure infections; cUTI, complicated urinary tract infections; DCGI, Drugs Controller General of India; EMA, European medicines agency EU ; FDA, Food and Drug Administration US; HABP, hospital acquired bacterial pneumonia; IV, intra-venous; MHRA, Medicines and Health Products Regulatory Agency UK; PMDA, Pharmaceuticals and Medical Devices Agency Japan; po, per os (oral administration); VABP, ventilator associated bacterial pneumonia.

### Cephalosporins

**Fig. 3.**
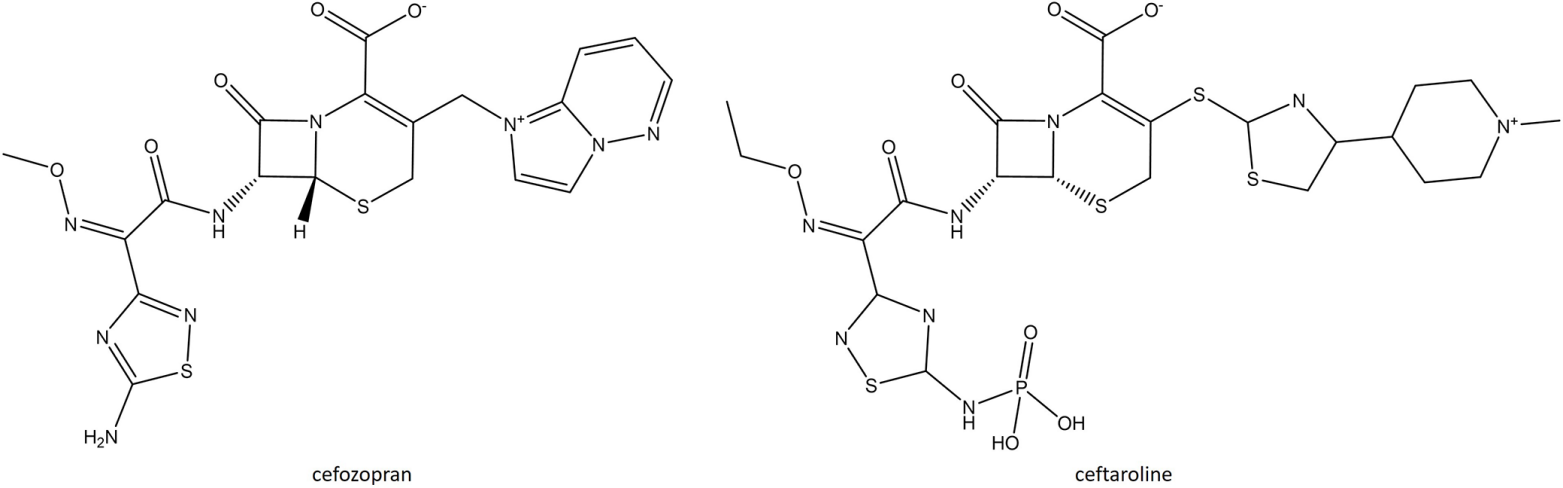
Structure of ceftaroline and its progenitor cefozopran.

One novel fifth-generation oxyimino-cephalosporin, ceftaroline fosamil (CFT), has been approved in the last decade and retains activity against MRSA and VRSA by binding covalently to PBP2A [123, 124]. It was developed by modifying the structure of the fourth-generation cephalosporin, cefozopran (Fig. 3). This includes a 1,3-thiazole ring at the 3-position of the cephalosporin nucleus as well as an oxime group in the C-7 acyl moiety and phosphono group, which together are responsible for the improved anti-MRSA activity and solubility of CFT [123]. CFT is marketed as a IV drug for use against *S. aureus* and other Gram-positive organisms and includes Gram-negative coverage. In phase III clinical trials, CANVAS 1 (NCT00424190) and CANVAS 2 (NCT00423657), CFT was compared with VAN/aztreonam (ATM) to treat complicated bacterial skin and skin structure infections (cSSSIs) caused by MRSA and other common cSSSI pathogens. In CANVAS 1, 703 patients suffering from cSSSIs were treated with either CFT (353) or VAN/ATM (349). The microbial cure rate for MRSA cSSSIs was 95.1 % for CFT and 95.2 % for VAN/ATM [125]. In CANVAS 2, clinical cure rates were similar between CFT and the comparator agents. MRSA cSSSIs were cured in 91.4 % of patients for CFT, and 93.3 % of patients treated with VAN/ATM. In both trials, similar rates of adverse events (AEs) were observed, with diarrhoea, nausea, headaches and pruritis the most frequently encountered. Overall, CFT demonstrated high clinical cure rates for both MRSA cSSSIs and cSSSIs caused by alternative pathogens whilst being well tolerated [126]. CFT was also evaluated in two FOCUS phase III studies (NCT00621504 and NCT00509106), assessing its potential in the treatment of community-acquired bacterial pneumonia (CABP). The clinical cure rates for *S. aureus* CABP was 72 % for CFT and 60 % for ceftriaxone and again it was well tolerated with minimal AEs [127]. Following the success of these trials, CFT gained US Food and Drug Administration (FDA) clinical approval in 2010 to treat CABP and acute bacterial skin and skin structure infections (ABSSSIs) and achieved European Medicines Agency (EMA) approval in 2012 for CABP and cSSSIs. Initial *in vitro* studies indicated that staphylococcal spp. acquired resistance at very low rates [128]. Despite this initial optimism, CFT-resistant strains of *S. aureus* have subsequently emerged and can be attributed to novel mutations in the CFT-binding pocket of PBP2A, including Y446N, E447K and E239K [129, 130].

### Quinolones

**Fig. 4.**
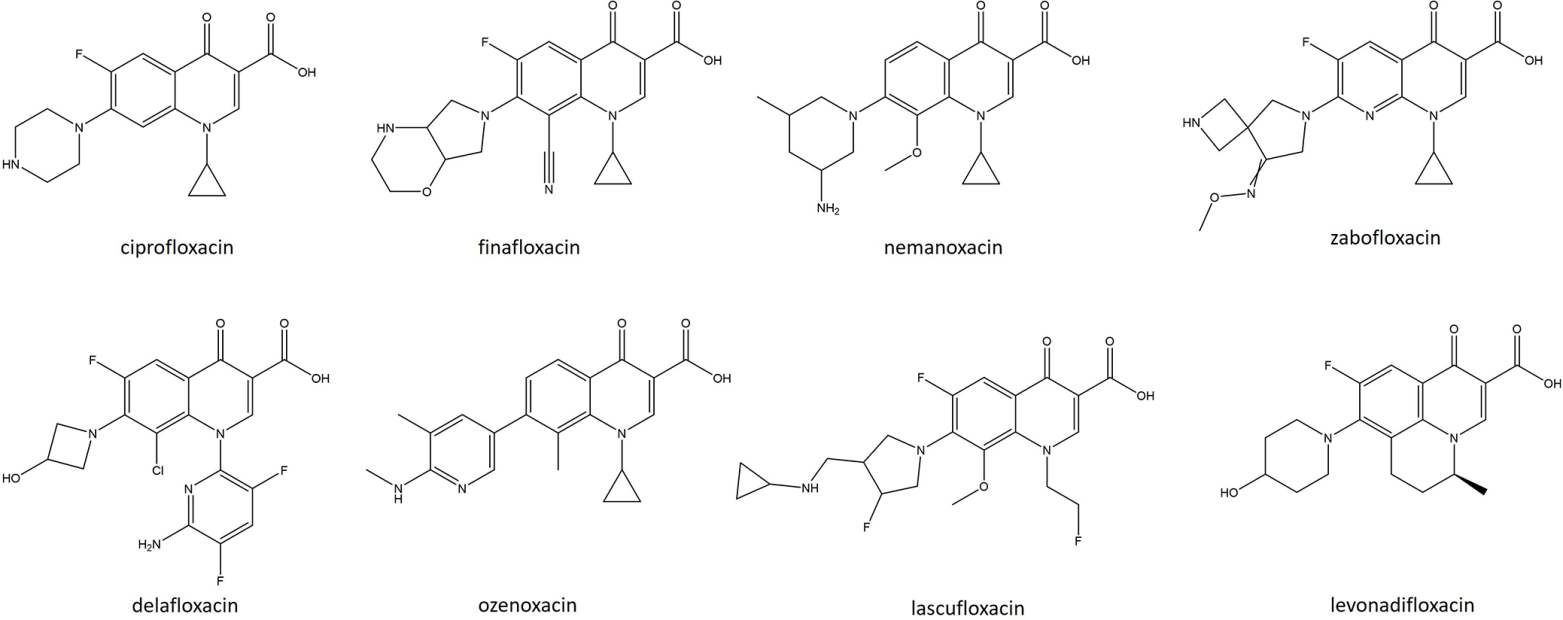
. Structures of recently approved quinolones (finafloxacin, nemanoxacin, zabofloxacin, delafloxacin, ozenoxacin, lascufloxacin, levonadifloxacin) and ciprofloxacin.

Seven new fluoroquinolone derivatives have been approved for clinical use since 2010. Despite initially being developed as anti-Gram-negative therapeutics, next-generation fluoroquinolones have been shown to have excellent anti-staphylococcal activity. The first of these, finafloxacin (FIN), is a novel fourth-generation fluoroquinolone harbouring an 8-cyano and 7-pyrrolo-oxazinyl substituent (Fig. 4) [131]. It has been shown to be effective in the clearance of infections caused by *S. aureus, Acinetobacter baumanii* and a range of other Gram-positive and -negative organisms [132]. Notably, this derivative was shown to have superior activity, when compared with CIP, in low-pH environments and infection-relevant conditions, likely due to its propensity to accumulate rapidly within bacterial cells combined with low rates of efflux [133–135]. In normal microbroth dilution assays FIN had a twofold lower MIC than CIP against community acquired MRSA [134]. When tested in synthetic urine, FIN demonstrated a 4–32-fold lower MIC than CIP against MRSA and methicillin-susceptible *S. aureus* (MSSA) [132]. FIN, as a topical formulation, has been evaluated in two randomized multicentre phase III trials for the treatment of otitis externa (NCT01535560, NCT01535599). Across both trials, pathogen eradication was achieved in 67 % of patients receiving FIN versus 13 % of patients receiving a vehicle control. The eradication rate for *S. aureus* was 89 % compared with 33 % for the placebo. Time to cure was also significantly shortened for patients receiving FIN, 3.5 days versus 6.8 days. The most frequently encountered AEs for topical FIN were nausea and pruritis of the ear canal, although this only occurred in 1 % of patients [131]. As a result, this topical suspension, under the trade name Xtoro, gained FDA and Health Canada approval as a drop suspension for the treatment of otitis externa in 2014. FIN has also completed a phase II trial (NCT01928433), administered by IV and orally for the treatment of complicated urinary tract infections (cUTIs). In this multicentre, randomized, double-blind, double-dummy, active-control trial FIN was administered for either 5 (FIN5) or 10 (FIN10) days compared with CIP for 10 days (CIP10). Clinical cure responses were observed for 70 % of FIN5 patients, 68 % of FIN10 patients and 57 % of CIP10 patients. FIN was generally well tolerated. Overall, 43.4, 42.7 and 54.2 % of patients in the FIN5, FIN10 and CIP10 groups experienced mild AEs. Together these results indicated that a 5 day course of FIN resulted in improved eradication and clinical outcomes compared with CIP [136]. Resistance studies involving this antibiotic against *S. aureus* are sparse, as the resistance breakpoints for this antibiotic have not yet been defined. However, *in vitro* studies involving finafloxacin noted that elevated MICs because of spontaneous mutation were rare. It has been noted that MIC measurements improve drastically when tested in acidic environments [131, 135].

Nemonoxacin (NEM) is a non-fluorinated C-8 methoxy quinolone targeting both DNA gyrase and topoisomerase IV (Fig. 4) [137]. C-8 methoxy substituents have previously been associated with an improved spectrum of activity and reduced mutant selection rates [74]. Indeed, NEM has been demonstrated to have broad-spectrum activity in both *in vitro* and *in vivo* studies against both Gram-positive and -negative pathogens but with a particular proclivity against MRSA and penicillin-resistant *S. pneumoniae* (PRSP). Removal of the fluorine side chain is believed to reduce toxicity [137, 138]. In a phase III, multicentre, randomized, double-blind, double-dummy, active-controlled, non-inferiority trial (NCT01529476), 500 mg of oral NEM was compared with 500 mg of LVX for the treatment of CABP [139]. Of a total of 527 patients, 356 received NEM and 171 received LVX, resulting in clinical cure rates of 94.3 and 93.5%, respectively. The incidence of AEs was also comparable between NEM (33.1 %) and LVX (33.3 %). Combined with the results of earlier phase II trials (NCT00434291 and NCT01537250), NEM reached non-inferiority versus LVX whilst maintaining a satisfactory safety profile [138, 139]. Following these trials, NEM gained Chinese approval in 2016 for the treatment of CABP and ABSSSIs and has since gone on to receive a qualified infectious disease product and It-track designation for CABP and ABSSSIs by the FDA. Due to its limited use, resistance has not currently been reported in the clinic, but *in vitro* studies have demonstrated resistance in *S. pneumoniae* strains harbouring three mutations within the quinolone resistance-determining regions (QRDRs): S82Y in *gyrA,* S494T in *gyrB* and P454S in *parC* . Thus, resistance to nemonoxacin is less likely to develop as resistance to other fluoroquinolones can occur through only two mutations in the QRDRs [140]. In another study involving clinical isolates of *S. pneumoniae, S. aureus, E. faecium* and *Enterococcus faecalis,* serial passage with nemonoxacin resulted in a fourfold increase in MIC against the *S. pneumoniae* isolate. However, this increase in MIC was found to be independent of mutations in the QRDRs. Contrastingly, the MICs against *S. aureus *, *E. faecium* and *E. faecalis* did not increase, demonstrating that nemonoxacin has a low potential for inducing resistance [141].

Zabofloxacin (ZBO) is a novel fluoronaphthyridone quinolone with a 7-pyrrolidone substituent (Fig. 4) that exhibits excellent *in vitro* broad-spectrum activity targeting both DNA gyrase and topoisomerase IV. It demonstrates impressive activity against a range of major respiratory pathogens, including fluoroquinolone-resistant *S. aureus *, *S. pneumoniae *, *Haemophilus influenzae* and *Moraxhella catarrhalis* [142]. In a phase III multicentre, double-blinded, randomized non-inferiority clinical trial (NCT01658020), oral administration of 367 mg (once daily for 5 days) of ZBO was compared with 400 mg of MOX (once daily for 7 days). The clinical cure rates were 88.2 % for ZBO and 89.1 % for MOX. No significant difference was observed, indicating that ZBO exhibited an equivalent clinical outcome for the treatment of chronic obstructive pulmonary disease (COPD). A sub-study in this trial also examined ZBO for the treatment of lower respiratory tract infection (LRTI) without the manifestation of chronic bronchitis. In this patient group the cure rate was 85.9 % for ZBO and 84.2 % for MOX, similarly without a significant difference being reached [143]. ZBO was approved for clinical use in the Republic of Korea in 2016 as an oral formulation for the treatment of acute bacterial exacerbation of COPD and later by countries in the Middle East and North Africa. Epidemiological studies in Egypt have shown that of 116 MRSA strains, 61.2 % were susceptible and 37.9 % were resistant, performing better than MOX, CIP and LVX. The resistant strains were found to have mutations in the QRDRs, namely S84L in *gyrA,* S80F in *parC* and P451S in *parE* [144].

Delafloxacin (DLX) possesses a unique, highly anionic scaffold that targets both DNA gyrase and topoisomerase IV of Gram-positive and -negative organisms with equal affinity (Fig. 4). DLX is the only non-zwitterionic fluoroquinolone currently available in the clinic [145, 146]. This molecule has an intriguing chemical structure, making it a weak acid, which stays uncharged in acidic environments, promoting transfer across the bacterial membrane and rapid accumulation within the cell [147]. Accordingly, DLX has increased activity in acidic environments such as the phagolysosome, within biofilms, abscesses and in the skin [147, 148]. It has broad-spectrum, bactericidal activity against multiple staphylococci (including MRSA), streptococci and enterococci, as well as many Gram-negative organisms [145, 146, 149]. A phase III multicentre, randomized, double-blind trial (NCT01984684) with VAN/ATM as a comparator was performed to determine the efficacy of DLX in the treatment of ABSSSIs. Eight hundred and fifty adults received either DLX (300 mg IV every 12 h for 3 days followed by a switch to 400 mg oral) or VAN/ATM (15 mg kg^−1^ for 5–14 days). Clinical objective responses were 83.7 % in the DLX treatment group and 80.6 % for the VAN/ATM combination. Treatment AE rates were similar between DLX and the comparator, but VAN/ATM was associated with a higher rate of drug-related discontinuation (1.2 vs 2.4 %). Thus, DLX reached non-inferiority for the treatment of ABSSSIs, which promoted its approval by the FDA in 2017 [150]. In contrast to other quinolones and due to the dual targeting ability of gyrase and topoisomerase, delafloxacin has increased stability and efficacy against gyrase and topoisomerase mutations [151]. Like NEM, double or triple mutations of the QRDR are required for the development of DLX resistance. However, DLX-resistant MRSA strains have been isolated from healthcare-associated infections. Genome sequence analysis of six DLX resistant isolates identified double mutations in *gyrA* (either S84L, Q88K or S85P) and a *parC* mutation (either S80Y/K, Q84G, or D36G) [152].

Ozenoxacin (OZE) is a novel non-fluorinated quinolone (Fig. 4). Like NEM, its non-fluorination grants a better safety profile than classical fluorinated quinolones, precluding the development of quinolone-induced chondrotoxicity and transdermal absorption [153]. Like DLX, OZE inhibits both gyrase and topoisomerase IV with equal affinity, conferring activity against CIP- and LVX-resistant *S. aureus, S. pyogenes, Staphylococcus epidermidis* and *Streptococcus agalactiae* [154, 155]. OZE's anti-staphylococcal activity has been further examined; in a study of 1031 *S *. *aureus* skin and soft tissue infection isolates, OZE exhibited an MIC_90_ of ≤0.05 µg ml^−1^, performing better than LVX [156]. Two multicentre, randomized, double-blind, vehicle-controlled phase III clinical trials (NCT01397461 and NCT02090764) have been performed to evaluate a 1 % topical OZE formulation for the treatment of impetigo. In the first trial, patients received either OZE, placebo or retapamulin (RET). Microbial clearance was 70.8 % for OZE versus 38.2 % for the placebo after 3–4 days and 79.2 versus 56.6 % after 6–7 days. OZE also contributed to more rapid microbial clearance than RET and was well tolerated [157]. In the second, 411 patients received either OZE or a placebo. OZE demonstrated superior cure rates after 5 days (54.9 versus 37.9 %). A notable superior microbial eradication was also noted after only 2 days (87.2 versus 63.9 %). It was similarly well tolerated, with only eight patients experiencing AEs, and only one potentially as a result of OZE administration [158]. Subsequently, this topical quinolone was approved in 12 European countries, the USA and Canada for the treatment of non-bullous impetigo in children. Like other dual targeting quinolones, OZE is less likely to result in the selection of resistant mutants. *In vitro* studies have shown that OZE maintains activity against isolates harbouring as many as four mutations in *gyrA/grlA* and is not affected by the efflux pump inhibitor reserpine [154].

Lascufloxacin (LSFX) is a novel 8-methoxy fluoroquinolone displaying a unique pharmacophore at the first and seventh positions of the quinoline nucleus (Fig. 4) [159]. Although it harbours some Gram-negative activity, its most potent activity was observed when tested against Gram-positive pathogens, including MRSA and *S. pneumoniae,* with an MIC_90_ of 0.06 µg ml^−1^ [159]. LSFX is well tolerated and achieves rapid distribution to the epithelial lining fluid and alveolar macrophages and achieves drug concentrations exceeding the MIC_90_ values for most MSSA and MRSA isolates [160]. In a double-blind, randomized comparative phase III study, LSFX (75 mg) was compared with LVX (500 mg) in patients suffering from CABP. The clinical efficacy rate following cessation of treatment was 96.0 % for LSFX and 95.8 % for LVX. The incidence of AEs was similar in both patient populations, 17.9 % for LSFX and 19.0 % for LVX with no serious AEs noted for either drug [161]. This drug was first approved for clinical use in Japan in 2019 where it has been used to treat CABP and other Gram-positive respiratory tract infections. Elevated MICs of 2 µg ml^−1^ (approximately a 16-fold increase) against LSFX have been reported and are due to double mutations in both *gyrA* and *parC*, namely S84L E88V or S84L E88K for *gyrA*, and S80F E84K for *parC*. These same mutations result in MICs of 32–128 µg ml^−1^ (a 64–256-fold increase) against CIP and LVX, therefore lascufloxacin shows increased activity against wild-type and mutated topoisomerase IV and gyrase enzymes and has a lower tendency to select for resistant mutants [159, 160, 162, 163].

The most recent quinolone to gain market approval is levonadifloxacin (LEV). This belongs to a new benzoquinolizine subclass of fluoroquinolones that is an arginine salt of the active *S*(−) isomer of the commonly prescribed topical fluoroquinolone nadifloxacin (Fig. 4). Unlike most archetypal fluoroquinolones, which preferentially target topoisomerase IV over DNA gyrase, LEV preferentially targets DNA gyrase [164, 165]. Due to the presence of a non-basic side chain, it remains unionized at an acidic pH, which allows for superior activity in acidic environments [166, 167]. It has broad-spectrum activity but shows particular potency against quinolone-resistant MRSA strains, hVISA, VRSA and DAP-resistant *S. aureus* [168]. It was evaluated in a phase III multicentre, randomized, open-label, active-comparator study with 500 subjects suffering from ABSSSIs (NCT03405064). Patients received either 1000 mg of oral LEV with 600 mg of oral LZD as a comparator, or 800 mg of IV LEV with 600 mg of IV LZD as a comparator. The clinical cure rate for LEV was higher for both the oral (91.0 vs 87.8 %) and IV (95.2 vs 93.6 %) formulations. At the lower bound of the 95 % confidence interval the treatment difference was greater than 15 %, meaning that both oral and IV LEV reached non-inferiority against oral and IV LZD. Approximately 30 % of patients tested positive for an MRSA infection, and LEV exhibited higher clinical cure rates in these patients (95.0 vs 89.3 %). AE rates were similar for IV treatment groups (20.8 vs 22.4 %, for LEV and LZD) and oral treatment groups (16.0 vs 13.5 %, respectively) and were all mild in nature [169]. This antibiotic recently gained approval and is available as an IV and oral tablet formation for use against ABSSSIs and diabetic foot ulcers in the USA. LEV is associated with resistance suppression properties. For example, while overexpression of the NorA efflux pump can lead to fluoroquinolone resistance, this has no effect on LEV activity, since it is not a substrate for NorA. Additionally, its higher affinity for DNA gyrase seems to come with a lower frequency of mutations [170]. This allows LEV to maintain activity against strains with multiple mutations in the QRDR with strains harbouring *gyrA* S84P and S85L, and *grlA* S80F mutations presenting with a LEV MIC of 2 µg ml^−1^ [171].

### Oxazolidinones

**Fig. 5.**
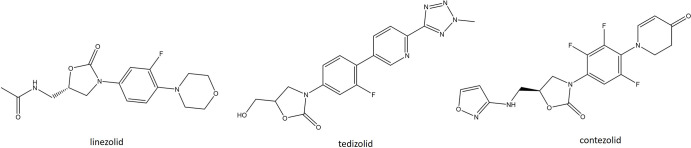
Structures of recently approved oxazolidinones (tedizolid and contezolid) compared with linezolid.

Two second-generation oxazolidinones have been approved clinically. Although structurally similar to LZD, tedizolid (TDZ) harbours two modifications that improve potency, increase binding affinity, and reduce its susceptibility to horizontally acquired resistance (Fig. 5). The first is the presence of a C-5 hydroxymethyl group replacing the acetamide group in LZD. The second is the addition of a C- and d-ring system, the incorporation of pyridine and tetrazole rings, respectively, enabling TDZ to form additional binding interactions within the upper region of the ribosomal peptidyl transferase centre [172, 173]. Together these modifications result in TDZ being four–eightfold more potent than LZD against MRSA and hVISA strains of *S. aureus *, vancomycin-resistant *E. faecium* (VRE) and streptococci [174]. Additionally, it has been shown to have improved oral bioavailability and a lower risk of drug interactions, culminating in an antibiotic with better safety and pharmacodynamics [175]. Two phase III clinical trials, ESTABLISH-1 and ESTABLISH-2, evaluated TDZ compared with LZD for the treatment of ABSSSIs. ESTABLISH-1 (NCT01170221) was a randomized, double-blind, noninferiority trial where either 200 mg of oral TDZ or 600 mg of oral LZD was administered every 12 h for 10 days [176]. ESTABLISH-2 (NCT01421511) was also a randomized, double-blind, noninferiority trial that compared patients receiving IV TDZ (200 mg for 6 days) with IV LZD (600 mg twice daily for 10 days) [177]. The result of both studies found TDZ reaching the non-inferiority threshold compared to LZD. Gastrointestinal (GI)-related AEs and myelotoxicity were also encountered less frequently in patients receiving TDZ [176, 177]. TDZ gained FDA approval in 2014 for the treatment of Gram-positive ABSSSIs. It is also being investigated in two phase IV trials for the treatment of *S. aureus* infections in cystic fibrosis (NCT02444234) and for the treatment of prosthetic joint infections (NCT03746327) and two phase II trials for the treatment of Gram-positive bone and joint infections (NCT03009045) and for the treatment of MDR *Mycobacterium tuberculosis* (NCT05534750). As with LZD, the spontaneous frequency of mutation against TDZ is low and the modified side chain at the C-5 position helps maintain activity, with MICs of 0.5–1 µg ml^−1^ against strains harbouring the horizontally transmissible *cfr* gene [178]. A recent study that sought to investigate the capacity of TDZ to select for cross-resistance to other antimicrobials found a A1345G single nucleotide variant in the *rpoB* gene after 10 days of serial passage that gave a TDZ MIC of 4 µg ml^−1^. This mutation also conferred elevated MICs against LZD, CHL, RET and quinupristin/dalfopristin [179]. Subsequent serial passaging experiments with TDZ and LZD showed an unchanged TDZ MIC of 0.5 µg ml^−1^ for MSSA ATCC 29213 but an elevated TDZ MIC from 0.25 µg ml^−1^ to 2 µg ml^−1^ for MRSA ATCC 33591. For LZD the MIC increased 32- and 64-fold for the MSSA and MRSA strains, respectively. The stepwise increase in TDZ MIC against MRSA ATCC 33591 was found to require multiple mutations in the 23S rRNA, but only single mutations were required to confer LZD resistance [180].

Contezolid (CZD) is a novel ortho*-*fluoro dihydropyridone second-generation oxazolidinone (Fig. 5). It differs from LZD by replacing the morpholine group with a piperidinone group [181]. This modification helps address the myelosuppression and monoamine oxidase (MAO) inhibition counterindications, which are known to be associated with LZD. LZD has been shown to bind and inhibit both A and B isoforms of the MAO enzyme and can therefore cause serotonin syndrome, especially when co-administered with serotonin re-uptake inhibitors, various inhibitors, or other MAO inhibitors [182]. CZD on the other hand has exhibited between 2- and 148-fold reduction in MAO-A and MAO-B inhibition *in vitro*. This was deemed to be of potential clinical significance, warranting the investigation of CZD in clinical trials [183]. The spectrum of CZD covers most Gram-positive pathogens, including MRSA, PRSP and VRE, although the potency of this drug is considered to have a similar efficacy to LZD. Furthermore, strains that are resistant to LZD are generally considered to be cross-resistant to CZD [181, 184, 185]. In a double-blind, multicentre, phase III study conducted in PR China, CZD was compared to LZD for the treatment of complicated skin and soft tissue infections (cSSTIs). In total, 596 patients received either oral CZD (800 mg every 12 h for 7–14 days) or oral LZD (600 mg every 12 h for 7–14 days). The clinical cure rate was 92.8 % for CZD and 93.4 % for LZD. The clinical AEs for CZD were comparable to those for LZD; nausea, vomiting, abdominal discomfort and increases in alanine aminotransferase, aspartate aminotransferase, blood uric acid and blood bilirubin levels were most the most frequently encountered AEs and were considered mild to moderate in their severity [184]. CZD was approved by the National Medical Products Administration of China in 2021 for the treatment of cSSSIs caused by a range of Gram-positive organisms, including MRSA, VRE, *S. pyogenes* and *S. agalactiae *. CZD is also under investigation in the USA in an effort to accelerate global approval; NCT03747497, a phase II trial for treatment of ABSSSIs, and NCT05369052, a phase III trial for the treatment of diabetic foot ulcers.

### Tetracyclines

Two tetracycline antibiotics entered the clinic in 2018. Eravacycline (ERV) is a broad-spectrum, fully synthetic fluorocycline that has been developed to treat various MDR organisms, such as MRSA, VRE and carbapenem-resistant *Enterobacteriaceae* (CRE) (Fig. 2). It was designed to overcome two mechanisms of TET resistance, namely drug efflux and ribosomal protection, by modifications to the C7 and C9 positions of the TET core [186]. Prior structural modifications of the C7 and C9 positions has led to the discovery and development of clinically important TET derivatives such as MIN and TGC [91, 187]. These antibiotics showed improved activity against tetracycline-specific efflux but remained susceptible to RPPs. The substitutions present in ERV are a pyrrolidine group on the C9 side chain and a fluoro group at C7. Together these improve the antibacterial potency against strains harbouring both TET efflux and RPP resistance determinants. ERV is largely unaffected by the presence of *tetM*, *tetK*, or *tetA* with a 4–16-fold increase in potency (MIC_90_ 0.016–0.05 µg ml^−1^) against *S. aureus *, *E. faecalis, Escherichia coli* and *Acinetobacter baumannii* compared to TGC. This *in vitro* activity has carried over to impressive *in vivo* efficacy in murine models, including a septicaemia model challenged with SHV-producing *E. coli *, and a neutropenic thigh model challenged with a *tetM*-producing *S. aureus* strain [186, 188]. Encouraging *in vitro* and *in vivo* data directed an examination of the clinical efficacy of ERV. The clinical trials IGNITE1 (NCT01844856) and IGNITE4 (NCT01844856) focused on the treatment of complicated intrabdominal infections (cIAI). IGNITE1 was a randomized, double-blind, double-dummy, comparative, noninferiority, multicentre, multinational trial consisting of 541 patients. Subjects received either 1 mg kg^−1^ IV ERV every 12 h or 1 g of IV ertapenem every 24 h, both for a median of 7 days. Isolated organisms were a mix of Gram-negative aerobes and Gram-positive anaerobes/aerobes. Clinical cure rates were 87.0 % for ERV and 88.8 % for ertapenem and reached non-inferiority criteria. There were three deaths in the ERV group and six in the ertapenem group but these were not believed to be a result of either drug treatment. More AEs occurred in the ERV group, specifically the incidence of nausea and phlebitis [189, 190]. IGNITE4 was a second randomized, double-blind, double-dummy, comparative, noninferiority, multicentre, multinational trial investigating cIAIs in a further 500 patients. Participants received either 1 mg kg^−1^ IV ERV every 12 h or 1 g of IV meropenem every 8 h, for 4–14 days. ERV similarly reached non-inferiority, with clinical cure rates of 91.2 % (ERV) and 90.8 % (meropenem). AEs were mostly gastrointestinal in nature and occurred in 37.2 % of the ERV group and 30.9 % of the meropenem group [191]. This was followed by the approval of this antibiotic by the EMA and FDA for the IV administered treatment of cIAIs. *S. aureus* resistance to eravacycline has not been reported, but MIC creeping verging on hetero resistance has been observed. Elevated MICs of 1–4 µg ml^−1^ were reported more frequently for MSSA strains. These were reversed when combined with efflux pump inhibitors, suggesting the involvement of efflux in this phenotype [192, 193].

Omadacycline (OMA) is a semisynthetic 9-aminomethylcycline derivative of MIN. Like the prototypical semisynthetic tetracyclines MIN and TGC, it harbours a C9 substitution to the tetracycline core (Fig. 2). Specifically, OMA has a an aminoethyl group present at the C9 position that overcomes TET resistance mechanisms commonly employed against MIN and DOX, including efflux and ribosomal protection [194]. These features allow OMA to maintain activity in the presence of *S. aureus* strains with *tetO* and *tetK* [195]. In addition, this structural modification increases the antimicrobial potency of this drug whilst reducing the unwanted side effects frequently encountered with TGC administration, such as nausea and emesis [195, 196]. OMA is an impressively broad-spectrum drug with coverage against aerobic and anaerobic Gram-positives, Gram-negatives and other atypical pathogens. For *S. aureus* it exhibits excellent *in vitro* activity against MRSA isolates with an MIC_90_ of between 0.25–0.5 µg ml^−1^ [193, 194, 197]. OMA has completed three phase III trials: OPTIC (NCT02531438) for the treatment of CABP and OASIS (NCT02378480) and OASIS-2 (NCT02877927) for the treatment of ABSSSIs. In OPTIC, a randomized, double-blind, multicentre study, 744 patients received either 100 mg IV OMA (initially twice daily but reduced to once daily) or 100 mg of IV MOX for 3 days. Clinical success at 5–10 days post-administration was 92.9 % for OMA and 90.4 % for MOX in the clinically evaluable population, reaching the non-inferiority threshold [198]. OASIS and OASIS-2 were randomized, double-blind, multicentre studies comparing the safety and efficacy of OMA to LZD. In OASIS, patients received IV OMA (100 mg twice a day for the first 2 days followed by 100 mg/day) or IV LZD (600 mg twice daily). Post treatment clinical success for the clinically evaluable (CE) population was comparable between OMA (96.3 %) and LZD (93.5 %). Success rates for *S. aureus* were also similar (OMA, 83.3 %; LZD, 83.4 %) [199]. In OASIS-2, patients received oral OMA (450 mg/day for days 1–2, then 300 mg/day thereafter) or oral LZD (600 mg twice daily). OMA again performed similarly to LZD, with success rates of 97.9 and 95.5%, respectively. In this study OMA was superior to LZD for the treatment of MSSA (82.7 vs 79.8 %) and MRSA (85.6 vs 79.4 %) [200]. In all phase III trials OMA was deemed safe and to have high tolerability, with only minor GI issues noted [198–200]. It also reached non-inferiority vs its comparator in all cases, leading the FDA to approve it for the treatment of CABP and ABSSSIs. The EMA approved OMA for use against ABSSSIs, requiring a further phase III CABP study (NCT04779242). OMA heteroresistance has been reported for clinical isolates *in vitro*. Isolates with elevated MICs of ≥1 µg ml^−1^ were reduced to as low as ≤0.03 when performed in the presence of PAβN or CCCP. The authors suggested that, as is the case with TGC, *tetK* overexpression was the culprit for the elevated MICs. However, further analysis indicated that the overexpression of two putative genes, RS01625 and RS00550, contributes to omadacycline heteroresistance. Next-generation sequencing also found that heteroresistant strains harboured mutations in fibronectin-binding protein (FnBP), which the authors concluded may impede the penetration of OMA [201].

### Pleuromutilins

**Fig. 6.**
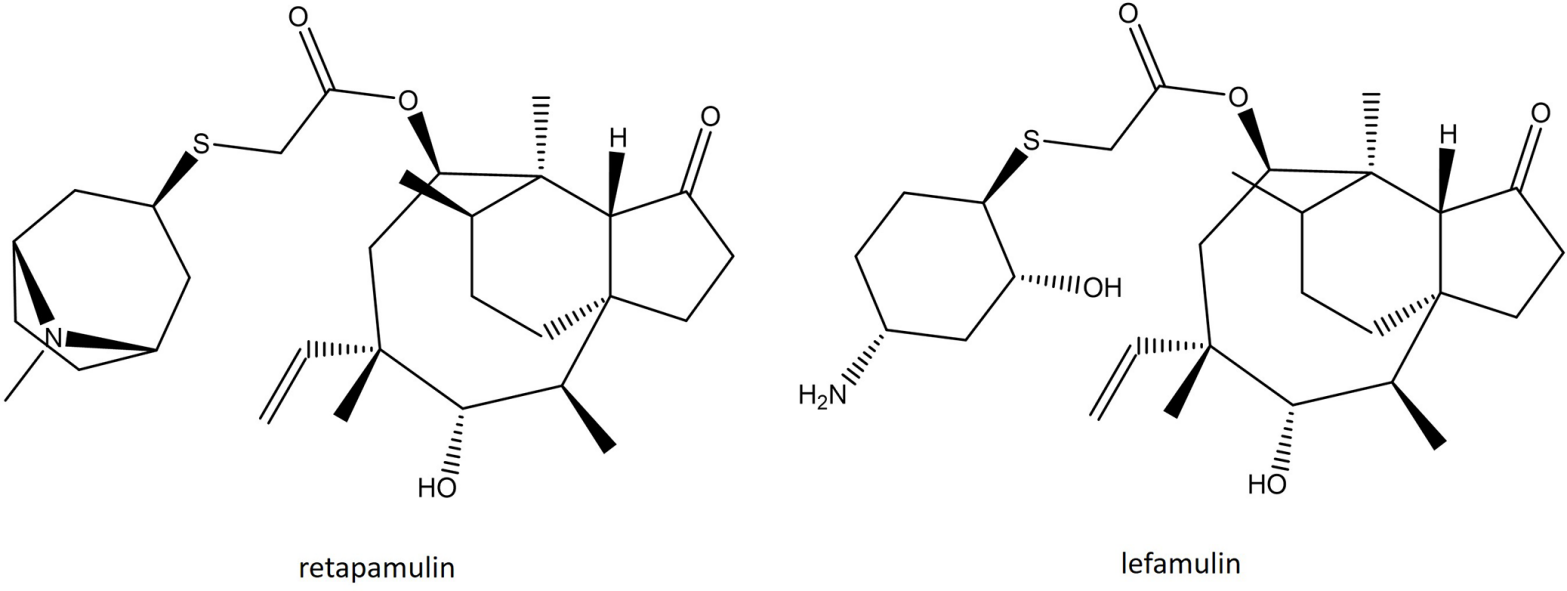
Structure of the recently approved pleuromutilin lefamulin compared with the first in class retapamulin.

Lefamulin (LEF) is a novel semisynthetic pleuromutilin consisting of a tricyclic mutilin core that helps facilitate an interaction with domain V of the 23S rRNA subunit through additional hydrophobic bonds and van der Waal forces (Fig. 6). In addition, this molecule possesses a large C14 extensional group that is the main driver of the pharmacodynamic and antimicrobial properties. This C14 side chain harbours a thioether bond that significantly improves solubility and metabolic stability, both of which enable LEF to be administered orally and intravenously [202, 203] and increases the number of hydrogen bonds with the target site, which lowers the impact of ribosomal mutations [202, 204]. LEF has a distinct mechanism of protein synthesis inhibition that involves binding at the A- and P-site, preventing peptidyl transferase reaction. It shares some similarities with the mechanism of action of oxazolidinones, but LEF employs an ‘induced-fit’ strategy to close the binding pocket of the ribosome. This mechanism is incredibly effective at preventing the formation of the first peptide bond, but once elongation has started, LEF is ineffective [202–205]. Thus, the activity of LEF is believed to be bacteriostatic against most bacterial species, with the exception of *Mycoplasma pneumoniae* [206]. Its spectrum of activity includes all Gram-positive aerobes except *E. faecalis,* as well as certain fastidious Gram-negative aerobes, such as *H. influenzae* and *M. catarrhalis*. Importantly, it retains activity against MRSA, VISA, hVISA and VRSA strains of *S. aureus* with an MIC_90_ of 0.12 µg ml^−1^, as well as MDR *S. pneumoniae* and VRE [207–209]. LEF has completed two phase III trials, LEAP 1 and LEAP 2. LEAP 1 was a multicentre, randomized, double-blind, double-dummy, active-controlled, parallel-group study (NCT02559310) that evaluated the efficacy of IV-to-oral LEF with MOX with or without LZD as a comparator in adults with CABP. A total of 551 patients received either 150 mg IV LEF (*n=*276) every 12 h, or 400 mg IV MOX (*n=*275) every 24 h. After six doses, this could be switched to 600 mg oral LEF every 12 h or 400 mg oral MOX every 24 h. Under this treatment regimen LEF reached non-inferiority to MOX, with an early clinical response of 87.3 vs 90.2 %, respectively. The rates of discontinuation due to AEs were 2.9 % for LEF and 4.4 % for MOX, with the most frequently reported AEs being hypokalaemia, insomnia, or pain during administration [210]. LEAP 2 (NCT02813694) was a further double-blind, double-dummy, parallel-group randomized clinical trial comparing oral LEF with oral MOX for the treatment of CABP. Patients received 600 mg oral LEF every 12 h for 5 days or 400 mg oral MOX every 24 h for 7 days. The clinical success response was 89.7 % (LEF) and 93.6 % (MOX) in the clinically evaluable population, reaching the non-inferiority threshold. AE rates were encountered in 12.1 % of the LEF group and 1.1 % of the MOX group, but were mild in nature [211]. Following these studies, LEF was deemed safe and effective for the treatment of CABP and gained approval by the FDA and EMA, making it the first pleuromutilin approved for systemic use. There is evidence of low frequency of spontaneous mutation (<10^−9^), making resistance developing during administration unlikely [202, 203]. Nevertheless, *in vitro* resistance has been described and is a result of 23S, *rplC* and *rplD* mutations, or acquisition of the Tn5406-encoded Vga(Av) or plasmid-borne Vga(A) [212].

### Lipoglycopeptides

In the last 9 years two synthetic lipoglycopeptides have been clinically used. Conventional glycopeptides inhibit late-stage peptidoglycan synthesis via their aglycone group, which forms five hydrogen bonds with the terminal d-Ala-d-Ala residue of lipid II [213]. Early work focussing on enhancing the activity of VAN suggested that lipid II binding could be enhanced through two mechanisms: (1) the formation of glycopeptide dimers that lock the binding pocket in the correct configuration and promote cooperative binding and (2) the anchoring of the glycopeptide to the membrane, which may help to maintain the drug in close proximity to its target and cause membrane disruption [214, 215]. Lipoglycopeptides retain the aglycone group and therefore conserve this primary mode of action. However, the addition of a lipophilic side chain linked by a disaccharide moiety anchors the molecule to the membrane and causes membrane-induced bacteriolysis [214].

**Fig. 7.**
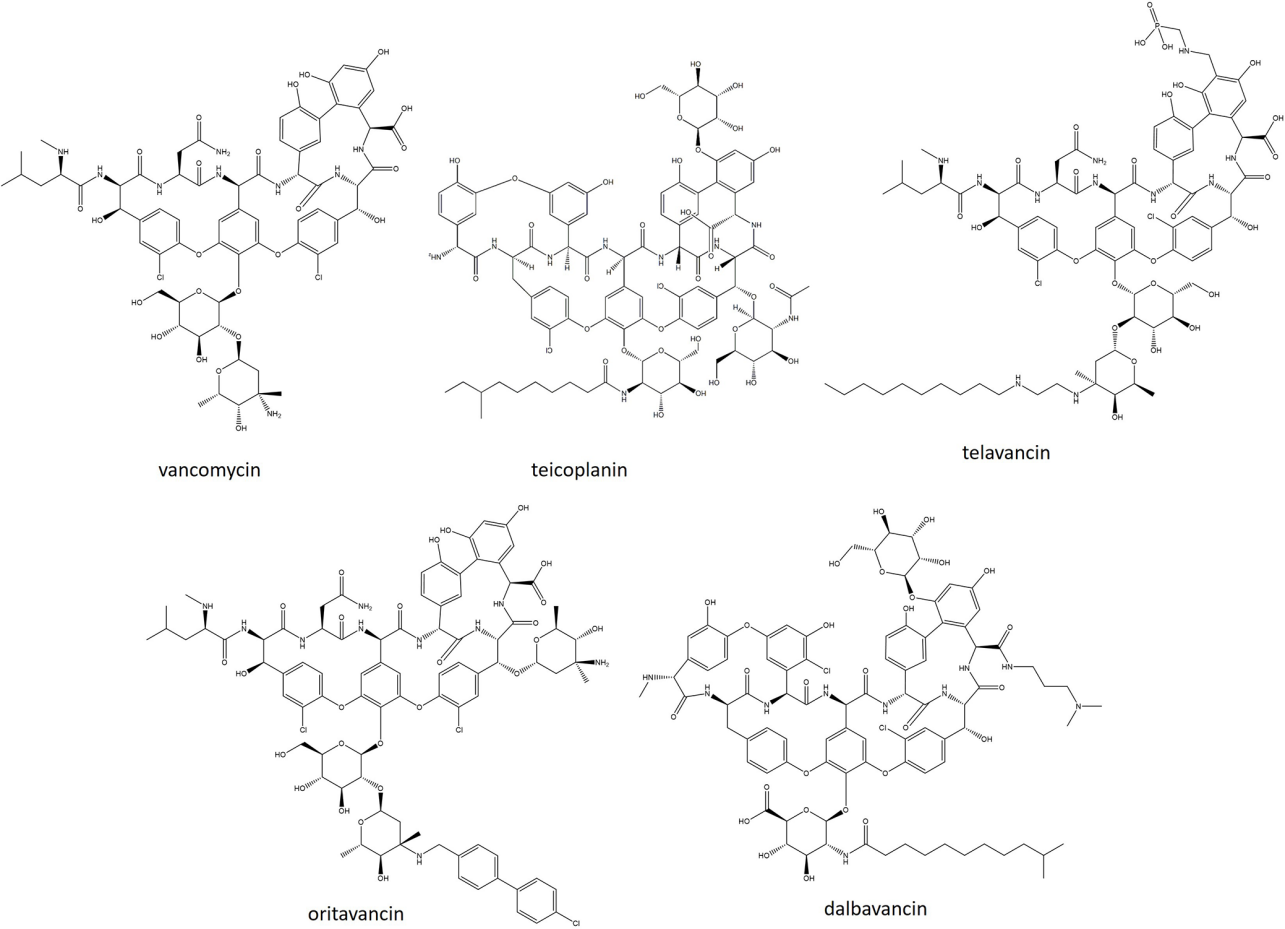
. Structures of recently approved lipoglycopeptides compared with vancomycin, teicoplanin and telavancin.

Oritavancin (ORI) is a chemically synthesized derivative of the naturally occurring lipoglycopeptide chloroeremomycin. It differs from chloroeremomycin through the addition of a chlorobiphenylmethyl side chain to the disaccharide moiety, which increases the amphipathicity of the molecule (Fig. 7) [216]. The presence of a 4-*epi-*vancosamine sugar in both ORI and chloroeremomycin contributes to dimer formation by putting the molecules in a back-to-back orientation [216, 217]. ORI has been demonstrated to disrupt membrane integrity and reverse membrane polarity [218, 219]. The lipophilic side chain also possesses an additional lipid II binding site that interacts with the pentaglycine bridge. This feature permits inhibition of both transglycosylase and transpeptidase reactions, explaining its improved activity against VAN-resistant strains [220]. Like all glycopeptides, ORI is only active against Gram-positive species. However, against VAN-susceptible staphylococci, streptococci and enterococci, it is more potent than VAN with an MIC_90_ of 0.06 µg ml^−1^ against *S. aureus *. ORI is the only lipoglycopeptide to be active against VRE harbouring *vanA* [221]. The suitability of oritavancin as a treatment option for ABSSSIs has been investigated in two phase III trials, SOLO 1 and SOLO 2, with VAN as a comparator. SOLO 1 was an international, randomized, double-blind study (NCT01252719) where participants were given a single IV dose of 1200 mg ORI followed by either IV placebo (*n*=475) or an IV dose of VAN (1 g or 15 mg kg^−1^) every 12 h for 7–10 days (*n=*479). All three efficacy end points (primary end point, assessed clinical cure and proportion of patients with a reduction in lesion size of ≥20 %) met the specified non-inferiority margin of 10 %. Overall, the frequency of AEs was similar between patient groups, but nausea was more common with ORI [222]. SOLO 2 was a global, multicentre, randomized, double-blind, comparative efficacy and safety study using the same dosing regimen as SOLO 1 (NCT01252732). Similarly, in this study all three efficacy endpoints met the 10 % non-inferiority margin. ORI was well tolerated with only mild AEs reported (nausea, vomiting, headache, cellulitis and increased alanine aminotransferase levels) [223]. Following the success of both trials, the FDA and EMA approved it for use against patients suffering from ABSSSIs. Resistance following clinical use has not been described for ORI, but elevated MICs of ≤16 µg ml^−1^ have been observed in a laboratory setting. This was achieved through mutations of the *vanS(B*) sensor gene*,* as well as hyperproduction of VanH, VanA and VanX. The presence of the *vanZ* gene also confers an MIC of 8 µg ml^−1^ through an unknown mechanism [224].

Dalbavancin (DAL) is a semi-synthetic derivative of the naturally occurring lipoglycopeptide A40926 [225]. The peptide carbonyl group present in the structure of A40926 is replaced by a 3,3-dimethylaminopropylamide group in DAL (Fig. 7) [226]. DAL has been shown to dimerize in solution but without the need for a ligand, due to the presence of an extended lipophilic side chain. However, unlike ORI, this type of dimerization is not believed to be co-operative [227]. Membrane anchoring has been demonstrated for DAL, which is hypothesized to increase the lipid II binding affinity, leading to increased antimicrobial potency [228]. Its spectrum activity is similar to that of other glycopeptides, displaying bactericidal activity against a variety of Gram-positive pathogens. It has 8–16-fold greater activity than VAN against staphylococci and streptococci. A recent examination of over 1100 MRSA isolates has shown the MIC_50_ of DAL to be 0.06 µg ml^−1^, compared with 1 µg ml^−1^ and 0.5 µg ml^−1^ for VAN and teicoplanin, respectively. Only three *S. aureus* isolates possessed MICs above the currently proposed FDA breakpoint [229]. In addition to its improved antimicrobial potency, DAL has an extended half-life, allowing it to be dosed once weekly [230]. Two phase III randomized, double-blind, multicentre, noninferiority studies clinical trials, DISCOVER 1 and DISCOVER 2 (NCT01339091 and NCT01431339), investigated the efficacy of DAL for the treatment of ABSSSIs. In both trials patients received either 1000 mg IV DAL on day 1 followed by 500 mg IV on day 8 or 1000 mg IV (or 15 mg kg^−1^) VAN every 12 h for 3 days. The VAN-treated group had the option to the switch, on day 3, to 600 mg orally administered LZD every 12 h for 12–14 days. Across DISCOVER 1 and 2, the primary endpoint indicated non-inferiority of DAL vs its comparator. Early clinical response rates were 79.7 % for DAL and 79.8 % for VAN–LZD. For patients infected with *S. aureus,* clinical success was seen in 90.6 % of the DAL treatment group and 93.8 % of the VAN–LZD group. The most commonly encountered AEs were nausea, diarrhoea and pruritis, but these were noted to be lower in the DAL treatment group [231]. DAL is also being investigated in several other trials, including a phase IV trial investigating osteomyelitis (NCT03426761), a phase IV trial investigating DAL over prolonged IV therapy (NCT03982030), a phase III trial investigating the suitability of DAL in the treatment of ABSSSIs in children (NCT02814916) and a phase II trial investigating *S. aureus* bacteraemia (NCT04775953). Resistance to DAL remains rare, occurring in only 1 % of isolates tested [232]. Similar to VAN, reduced DAL susceptibility is seen in hVISA and VISA strains. DAL does not retain activity against strains harbouring VanA or VanB, thus VRSA strains are similarly described as DAL-resistant [233, 234]. A recent case study of a patient with end-stage renal disease that developed *S. aureus* infective endocarditis found the causative MRSA isolate to be resistant to VAN, DAP and DAL due to *walk* and *scrA* mutations [235].

## Anti-staphylococcal compounds in clinical development

Here we review anti-staphylococcal compounds currently engaged in phase III and phase II clinical trials, representing novel, first-in-class antibiotics, heterodimer antibiotic conjugates, peptidomimetic AMPs and improved derivatives of existing antibiotics. Antibiotics that have since been dropped by pharmaceutical companies, or that have not had an update for the last 5 years, are not included for comment. Also not included are alternatives to antibiotics, such as vaccines, neutralizing antibodies, bacteriophage and anti-virulence strategies. Recent reviews on alternatives to antibiotics currently in the clinical pipeline are provided by Ghosh *et al.,* Douglas *et al.* and Czaplewski *et al.* [236–238]

### Iclaprim: a cautionary tale

**Fig. 8.**
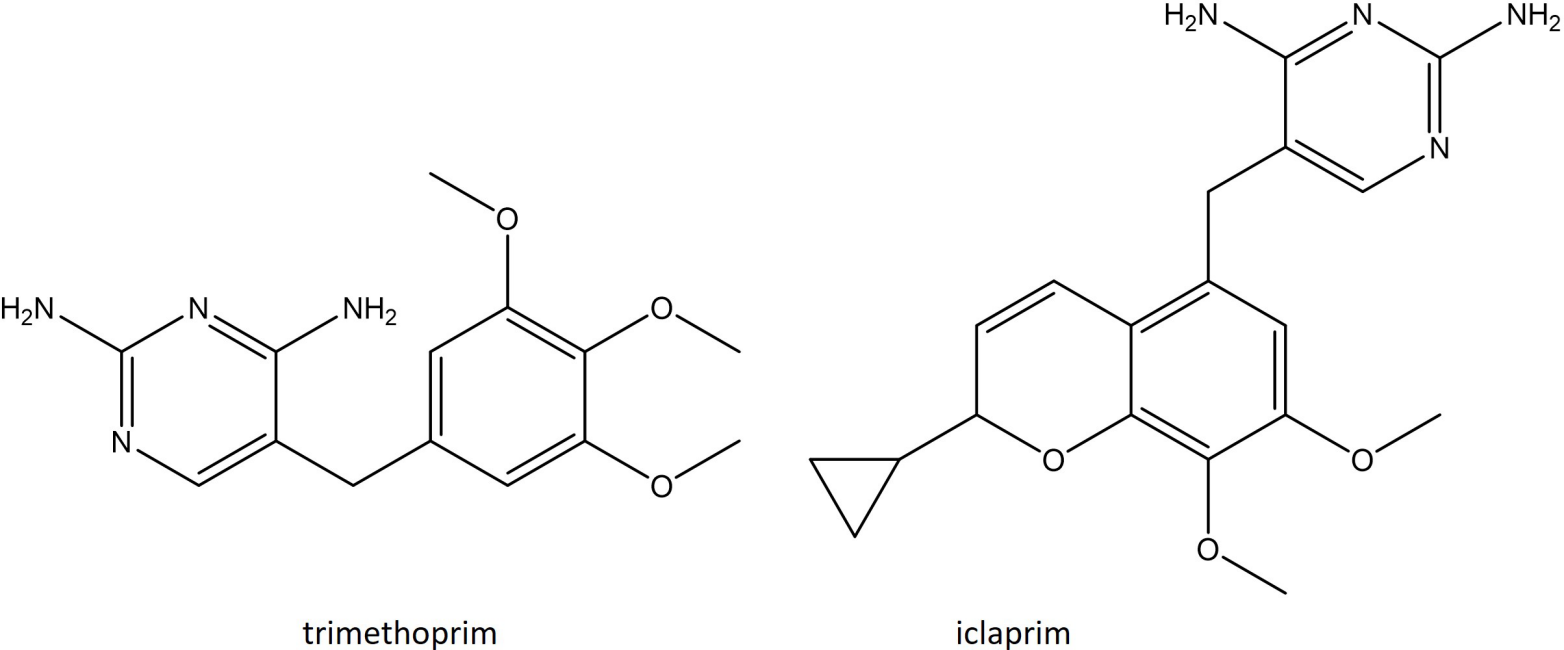
Structure of iclaprim compared with the first-in-class trimethoprim.

Iclaprim is only the second DHFR inhibitor that has reached the new drug application (NDA) stage of clinical development (Fig. 8). Iclaprim was developed following an in-depth enzymatic, direct binding and x-ray crystallographic analysis of compound interaction with *S. aureus* TMP-resistant DHFR (F98Y variant), as well as the *S. pneumoniae* TMP-resistant DHFR (I100L variant) isolates [239]. Enhanced hydrophobic interactions of iclaprim within the substrate-binding pocket of DHFR are essential for increased affinity and antibacterial activity, which correspond to a 20-fold increase in potency compared with TMP [239], allowing for the administration of iclaprim without the need for a sulphonamide. Therefore, sulphonamide-associated safety issues such as rashes, hypersensitivity, blood dyscrasias and life-threatening hyperkalaemia, can be avoided [240]. This drug has had a long and arduous development process spanning more than 20 years. Initially developed by Roche, Arpida subsequently obtained exclusive ownership and submitted an NDA for iclaprim in 2009 that was ultimately rejected, with the FDA citing that the most recent phase III trial did not demonstrate the efficacy of iclaprim in the treatment of ABSSSIs within an acceptable non-inferiority margin. Iclaprim changed hands several times before Motif Bio became owners and completed two phase II trials, REVIVE 1 (NCT02600611) and REVIVE 2 (NCT02607618), comparing the efficacy of iclaprim vs VAN for the treatment of ABSSSIs [241, 242]. A second NDA was submitted in 2018 but this was again rejected, citing additional toxicity testing requirements. Unfortunately, not gaining NDA approval resulted in the collapse of the company and forced this promising antibiotic to be shelved for the foreseeable future.

### Phase III clinical trials

**Fig. 9.**
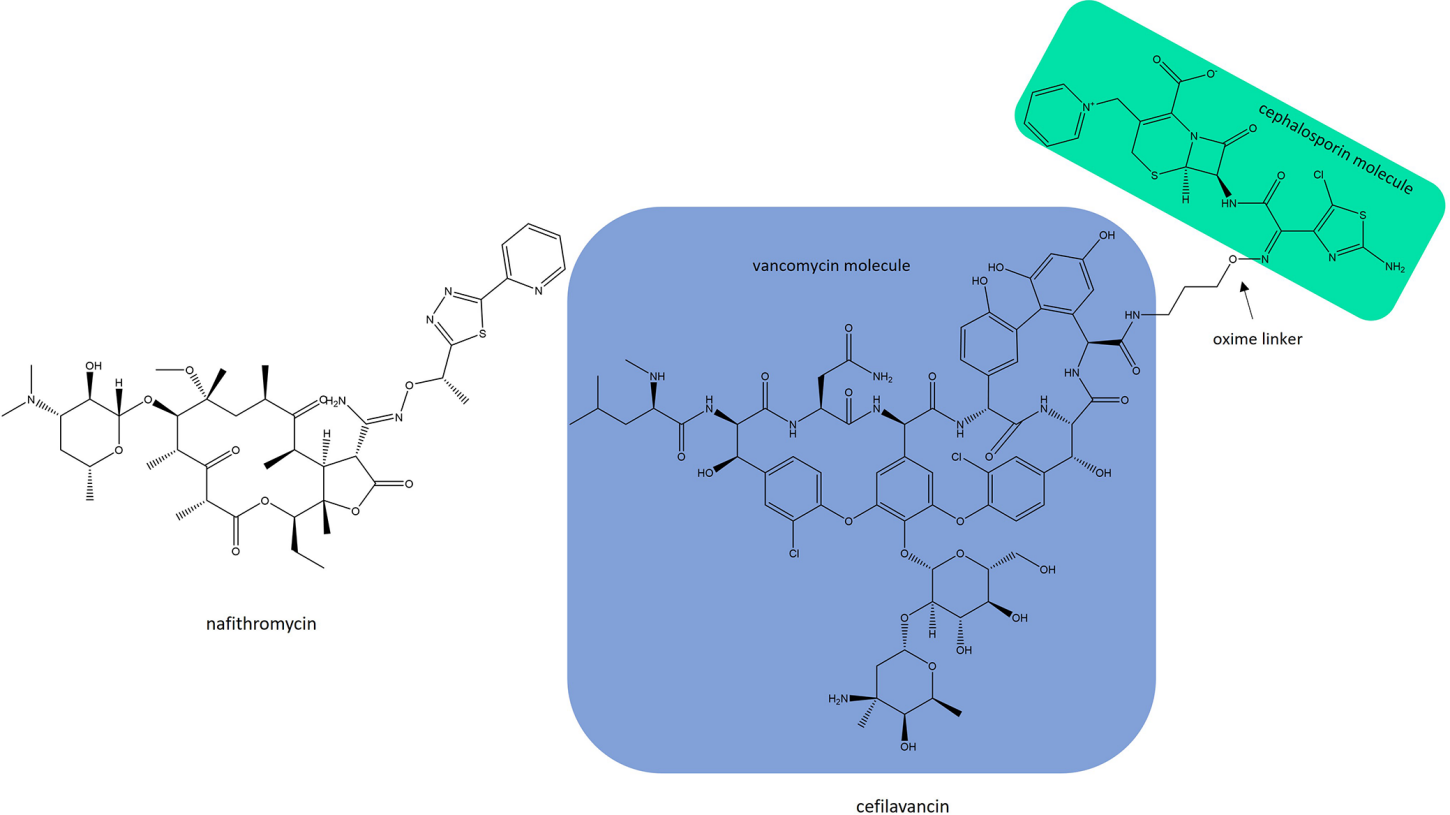
Structures of phase III anti-staphylococcal compounds.

There are two antibiotics in phase III clinical development for the treatment of *S. aureus* infections (Table 3). Nafithromycin is a novel lactone ketolide derived from ERY that lacks the l-cladinose moiety present in earlier macrolides, instead having a ketone in its place (Fig. 9). This increases the number of binding positions with the ribosome, which allows nafithromycin to maintain activity against certain macrolide-resistant organisms [243]. Nafithromycin differs from other members of the ketolide class, namely solithromycin and telithromycin (TEL), by having a lactone group at positions C11 and C12 instead of a carbamate. Furthermore, this lactone group is attached to a 2-pyridine-1,3,4-thiadiazole side chain, which enables nafithromycin to demonstrate activity against TEL-resistant *S. pneumoniae,* as well as promote oral availability [244]. A global surveillance study has shown that nafithromycin has improved activity against *S. aureus* compared with ERY, clindamycin (CLI) and TEL. Nafithromycin exhibited MIC_50/90_ of 0.06/>2 µg ml^−1^ against 716 MRSA and MSSA isolates. The MIC_50/90_ against ERY-resistant strains with an inducible phenotype was 0.06/0.06 µg ml^−1^. However, nafithromycin exhibited limited activity against TEL-resistant and CLI-resistant strains where the resistance phenotype was constitutively expressed. Nevertheless, the study highlighted the improved activity of nafithromycin where only 11.4 % of strains were resistant compared with 37.2 % for ERY[244]. Having shown favourable clinical outcomes in a phase II, randomized, double-blind, comparative study evaluating oral nafithromycin vs oral MOX for the treatment of CABP in adults (NCT02903836), a phase III trial has now been initiated. This will be a randomized, multicentre, double‐blind, comparative study (CTRI/2019/11/021964) investigating the oral administration of nafithromycin vs MOX in CABP patients in India.

**Table 3.** Antibiotics in phase III clinical trials. Antibiotics are described by company, drug class, route of administration and indications

Antibiotic	Company	Drug class	Route of administration	Indication	Trial no.
Nafithromycin	Wockhardt	Macrolide	po	CABP	CTRI/2019/11/021964
Cefilavancin	Theravance/R-pharm	Glycopeptide-cephalosporin heterodimer	IV	cSSSIs	

CABP, community acquired bacterial pneumonia ; cSSSI, complicated skin and skin structure infections; IV, intra-venous; po, per os (oral administration).

Cefilavancin (TD-1792) is a VAN–third-generation cephalosporin conjugate. The VAN molecule is covalently bonded from its terminal carboxyl group to the cephalosporin lactam amine group via a stable oxime linker (Fig. 9) [245]. The two in one nature of cefilavancin enables it to target both d-Ala-d-Ala-containing peptidoglycan precursors, as well as the active sites of PBPs, which culminates in a dual-pronged mode of action. Promisingly, the activity of cefilavancin is largely unaffected by pre-existing resistance mechanisms [246]. Moreover, the antibacterial effect could not be recreated by using individual components, consistent with a multivalent mechanism [247]. This molecule exhibits potent *in vitro* activity against *S. aureus *, with MSSA strains exhibiting an MIC_90_ of 0.015 µg ml^−1^ and MRSA/hVISA strains an MIC_90_ of 0.03 µg ml^−1^. Time–kill studies also showed bactericidal activity of cefilavancin at concentrations of ≤0.12 µg ml^−1^ and a post-antibiotic effect of >2 h [246]. In phase I studies, plasma concentrations following IV injection of cefilavancin at 2 mg kg^−1^ were consistently maintained for 24 h above the MIC at which 100 % of MRSA strains were killed [246]. A randomized, double-blind, active-controlled, phase II trial (NCT00442832) exploring the safety and efficacy of cefilavancin in patients with cSSSIs caused by suspected or confirmed Gram-positive organisms was conducted [247]. The clinical cure rates were 91.7 % for cefilavancin and 90.7 % for VAN. AEs occurred at similar frequencies, except for itching, which was more common in the VAN treatment group. Currently, the manufacturer is in the process of launching a phase III trial for the treatment of cSSSIs throughout Russia and Georgia.

### Phase II clinical trials

A total of nine anti-staphylococcal compounds are currently in phase II clinical trials (Table 4). OP-145 (previously known as P60.4Ac) is a synthetically modified LL-37 antimicrobial peptide (AMP) that was constructed to maintain its Gram-positive/-negative antimicrobial activity whilst minimizing the secondary pro-inflammatory response of LL-37 [248]. It contains several structural modifications, including a more favourable amphipathic helix formation as well as N- and C-terminal alterations to protect against proteolytic degradation, which improves its *in vivo* stability. OP-145 has been shown to be effective at killing MRSA in both planktonic and biofilm *in vitro assays,* as well as an MRSA-infected thermally wounded human skin equivalent model. OP-145 also shows no toxicity and displays higher or equal activity compared to LL-37 [249]. Preliminary investigation of OP-145 as a local topical therapeutic has shown that it retains activity within formulations suitable for skin and nasal mucosa administration [250]. This helped to spur the clinical development pathway with a randomized, double-blinded, placebo-controlled multicentre phase II study that investigated the suitability of OP-145 as an ototopical solution for the treatment of chronic suppurative otitis media (CSOM). In this study, the application of OP-145 to the ear canal of patients was safe and well tolerated with only mild to moderate AEs reported that were most likely unrelated to the study treatment. OP-145 treatment also demonstrated a higher treatment success compared to the placebo group (47 vs 6 %) [251]. OP-145 has also undergone follow-up modifications, yielding a new peptide SAAP-148 that demonstrated a superior activity profile. This peptide is currently under pre-clinical investigation [252, 253]. The discovery and improved potency of SAAP-148 may cause the developer to call a halt to the development of OP-145 and instead focus on SAAP-148.

**Table 4.** Antibiotics in phase II clinical trials*.* Antibiotics are described by company, drug class, route of administration and indications. Previous developmental names are indicated in parentheses

Antibiotic	Company	Drug class	Route of administration	Indication	Trial no.
OP-145 (P60.4Ac)	OctoPlus	Antimicrobial peptide	Ototopical solution	Chronic otitis media	ISRCTN12149720
LTX-109 (Lytixar)	Lytix Biopharma	Antimicrobial peptide	Topical	Impetigo	NCT04767321
Nilofabicin (CG400549)	Crystal Genomics	Benzyl-piridinone^†^	po	ABSSSIs	NCT01593761
Pravibismane (MBN 101)	Microbion corporation	Bismuth-ethanedithiol*	Topical	Diabetic foot infections	NCT05174806
XF-73 (exeporfinium chloride)	DestinyPharma	Di-cationic porphyrin*	Nasal gel/topical	Nasal decolonization	NCT03915470
Afabicin (Debio-1450)	Debiopharm	Pyrido-enamide^†^	IV, po	ABSSSIs, BJIs	NCT02426918 NCT01519492 NCT03723551
Rifaquizinone (TNP-2092)	TenNor Therapeutics	Rifamycin-quino lizinone hybrid	IV,po	PJIs, ABSSSIs	NCT03964493
Brilacidin	Innovation Pharmaceuticals	Defensin mimetic*	IV	ABSSSIs	NCT02052388
Delpazolid	LegoChem Biosciences	Oxazolidinone	po	Bacteraemia	NCT05225558

*denotes a first in class antibiotic

^†^denotes a first in class antibiotic with a novel target

ABSSSI, acute bacterial skin and skin structure infections; BJI, bone and joint infections ; BSI, bloodstream infections; IV, intra-venous; PJI, prosthetic joint infections; po, per os (oral administration).

LTX-109 is another naturally inspired peptidomimetic therapeutic. It was designed through initial identification of essential residues within lactoferricin B, through a full alanine scan, and subsequent substitution of individual residues. The resulting structure of LTX-109 contains cationic and lipophilic groups as well as nongenetically encoded amino acids, a modified tryptophan residue and an ethyl phenyl group at the C-terminus [254]. It is broad spectrum and displays a rapid bactericidal effect against both MRSA and VRSA strains, with an MIC range of 2–4 µg ml^−1^. Like OP-145, it also maintains its antimicrobial activity in the presence of host endoproteases [254, 255]. Thus far, LTX-109 has not been associated with cross-resistance to other natural and synthetic AMPs and has been shown to have a low propensity for the development of resistance, likely related to its rapid accumulation within bacterial membranes [256, 257]. An initial phase I/IIa clinical trial assessing nasal decolonization in MRSA and MSSA carriers has demonstrated the clinical efficacy of LTX-109 (NCT01158235). In this study, patients were treated for 3 days with either placebo, 1%, 2 % or 5 % LTX-109. In the 2 and 5% treatment groups, a statistically significant decrease in *S. aureus* burden was observed, measured by colony-forming units (c.f.u.) recovered from nasal swabs. Systemic levels of LTX-109 was very low and no safety concerns were encountered following a 9 week period [258]. A further phase II, double-blind, placebo-controlled, randomized study (NCT04767321) designed to evaluate the safety, tolerability and exploratory efficacy of 3 % LTX-109 gel in persistent *S. aureus* carriers was completed in 2021, but the results have not been published.

**Fig. 10.**
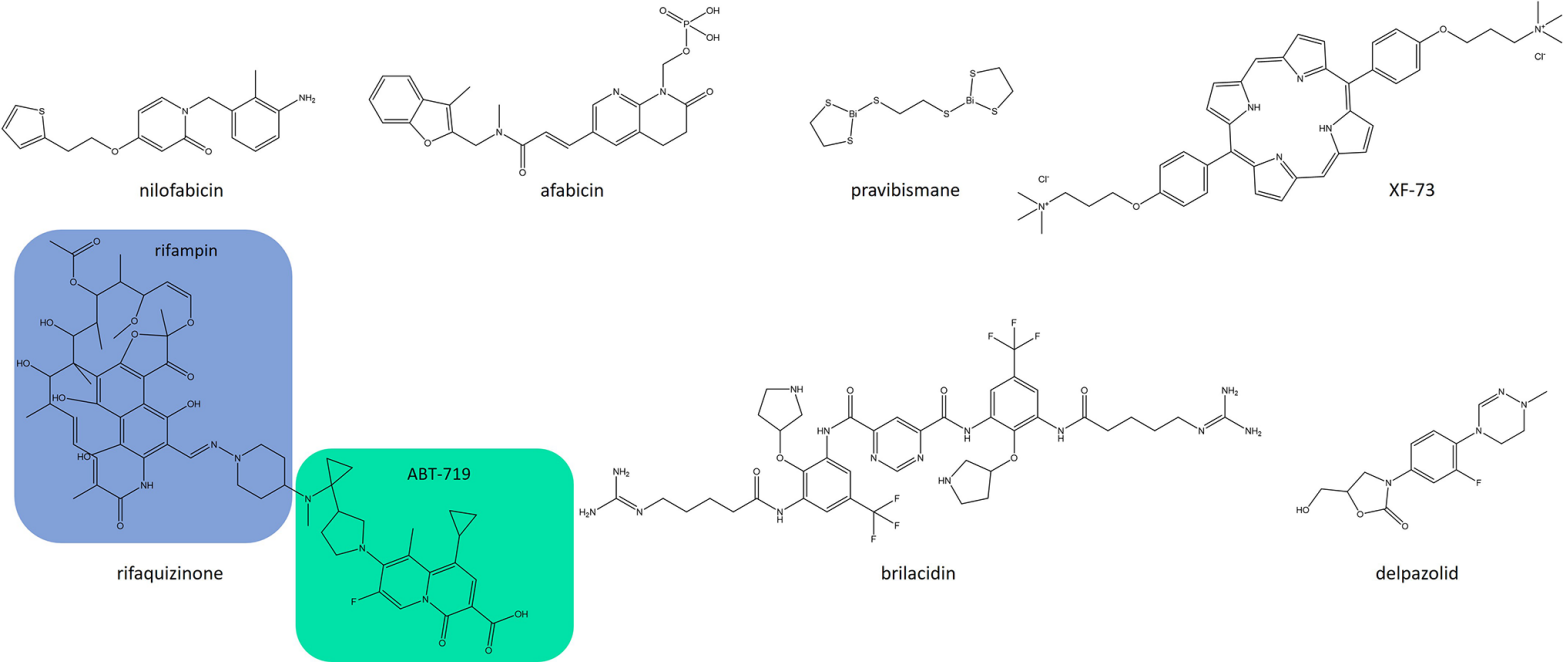
*.* Structures of phase II anti-staphylococcal compounds.

Nilofabicin (CG400549) is a novel benzyl-pyridone FabI inhibitor (Fig. 10). The bacterial fatty acid biosynthesis (FAS) pathway provides essential precursors for the assembly of numerous important cellular components, including phospholipids, lipoproteins and teichoic acids. The final step of this pathway is catalysed by the enoyl-ACP reductase (FabI), which is responsible for the reduction of enoyl-ACP derivative. This is an important reaction, as the reduction of enoyl-ACP derivatives dictates the ratio of saturated : unsaturated fatty acids, coordinates fatty acid and phospholipid synthesis and play a central role in the final stages of fatty acid elongation [259]. In *S. aureus,* FabI is the only enoyl-ACP reductase and is therefore essential [260]. Nilofabicin has an impressive spectrum of activity against MDR *S. aureus *, which includes MRSA and MSSA, as well as quinolone-resistant isolates. Against a panel of clinical isolates, it demonstrated an MIC_90_ of 0.25 µg ml^−1^. It has proven to be effective in eradicating systemic infections in mice, and through time–kill analysis has been shown to be a bacteriostatic agent. A FabI-overexpressing *S. aureus* strain resulted in higher MICs against nilofabicin. Higher MICs were also seen during *in vitro* selection of nilofabicin-resistant mutants, revealing a unique FabI (F204L) mutation, which confirmed FabI as the primary antibacterial target [261]. The pharmacokinetics, tolerability and efficacy of nilofabicin were demonstrated in a recent phase IIa open label exploratory study in test subjects with complications (NCT01593761).

Afabicin desphosphono (Debio 1452; AFN-1252) and its prodrug afabicin (Debio 1450; AFN-1720) is a second FabI inhibitor currently in phase II clinical development (Fig. 10). It is structurally different from nilofabicin, composed of a 3-methylbenzofuran and an oxotetrahydronaphthyridine linked through a *N-*methylpropenamide group. Afabicin is a narrow-spectrum drug, with potent anti-staphylococcal activity – an MIC_90_ of 0.015 µg ml^−1^ against clinically relevant MSSA and MRSA isolates [262] – and has proven to be more effective than LZD in mouse models of septicaemia [263]. These authors also noted a low dose-dependant propensity for resistance development, with an average frequency of 4.8×10^−10^. The FabI mutation M99T corresponded to a twofold increase in afabicin MIC but had no effect on the activity of triclosan. A second Y147H mutation that mapped to the FabI active site corresponded to an 8- to 16-fold increase in afabicin MIC, and a 64- to 128-fold increase in triclosan MIC [263]. An open label phase II proof-of-concept study (NCT01519492) to evaluate the efficacy of a daily dose of 400 mg afabicin in the treatment of ABSSSIs caused by staphylococci was completed in 2012. In this study the early response rate at day 3 was 97.3 %. More specifically, 82.9 % of patients had a ≥20 % decrease in erythema area, and 77.9 % of patients had a ≥20 % decrease in the area of induration. Afabicin was generally well tolerated, with drug-related AEs mostly mild or moderate in nature [264]. A second phase II multicentre, randomized, parallel-group, double-blind, double-dummy study has also recently been completed that investigated the efficacy, safety and tolerability of IV afabicin vs IV VAN with oral LZD in the treatment of ABSSSIs (NCT02426918). Both low- and high-dose afabicin reached non-inferiority and were well tolerated. The most common AEs reported were headache and nausea [265]. Debiopharm has recently started recruiting for another phase II randomized, active-controlled, open-label study (NCT03723551) to assess the safety and tolerability of afabicin in the treatment of subjects with bone and joint infection due to *S. aureus.*

Many metal-based co-ordinated complexes have played key roles in human medicine; salvarsan, an organoarsenic compound used for the treatment of syphilis and gold-complexed auranofin for rheumatoid arthritis therapy are some notable examples. More recently, bismuth-containing complexes have emerged as attractive antimicrobial candidates. Pravisbismane is a bismuth–thiol compound that is currently under development for the topical treatment of diabetic foot infections (Fig. 10). Although its exact mechanism of action and structure are undisclosed, the developer Microbion corporation states that pravibismane “is the first in a new class of bioenergetic inhibitors”https://microbioncorp.com/_news/microbion-corporation-announces-generic-name-for-lead-antimicrobial-compound-pravibismane. It serves to inhibit membrane-dependent ATP biosynthesis, resulting in a cessation of downstream processes. A study investigating the antibacterial effects against *E. coli* found that pravibismane acts rapidly, within 30 min, to completely shut down cell growth, rather than cause cell lysis, in a way that is reminiscent of non-specific cell-damaging agents such as detergents [266]. This study also revealed the effect of pravibismane on the bioenergetics of the cell membrane. Through the use of the membrane potential-sensitive dye DiBAC4(5), pravibismane was shown to depolarize the bacterial cells in a dose-dependent manner [266]. Furthermore, a luciferase-based assay determined that incubation with pravibismane caused a decrease in luminescence, showing that intracellular ATP levels were depleted [266]. This was unlike other test antibiotics, which had limited to no effect on intracellular ATP. Although performed on *E. coli *, it is likely that similar effects are observed with *S. aureus* [266]. A phase II randomized, open-label, controlled, multicentre study to assess the safety, tolerability and efficacy of topically applied pravibismane in patients with diabetic foot infections is currently in the recruitment process (NCT05174806).

Naturally occurring porphyrins such as haeme have long been noted for their antibacterial activity. Much of this antibacterial activity is based on their ability to catalyse peroxidase and oxidase reactions, resulting in the formation of reactive oxygen species (ROS) [267]. XF-73 is a leading drug candidate being developed by Destiny Pharma and is based on the porphyrin moiety (Fig. 10). First synthesized in 2009, XF-73 is a di-cationic porphyrin that has been shown *in vitro* to kill MSSA, MRSA, DAP-resistant, LZD-resistant and VISA strains. XF-73 exhibited an MIC of 1 µg ml^−1^ against *S. aureus* strain SH1000, portraying a more rapid killing kinetics than all other comparator agents. Investigation into the mechanism of action of XF-73 found that it interacted with the bacterial membrane, but rather than causing lysis, it instead causes membrane perturbation, resulting in the intercellular leakage of ATP and K^+^. It was also noted that a 10 min exposure of *S. aureus* to XF-73 caused a complete inhibition of DNA, RNA and protein synthesis [268]. The clinical investigation of XF-73 began with a phase I (NCT01592214) randomized, open-label study that tested the suitability and tolerability of two nasal formations: a 0.5 mg g^−1^/2 % gel and a 2 mg g^−1^/2 % gel. No systemic absorption of XF-73 was detected, and treatment was associated with a rapid reduction in *S. aureus.* Similarly, XF-73 was well tolerated, with only rhinorrhoea and nasal dryness noted as AEs [269]. A further multicentre, randomized, placebo-controlled, phase 2 study (NCT03915470) assessed the effect of nasal XF-73 on the nasal burden of *S. aureus* in patients undergoing cardiac surgery. After three applications of XF-73 over a period of 24 h, a greater reduction in nasal *S. aureus* c.f.u. was noted vs the placebo. No application site reactions or other treatment-emergent AEs were noted [270]. XF-73 is also in pre-clinical development for the treatment of superficial skin infections in PR China, the USA and the EU.

Rifaquizinone (TNP-2092) is heterodimer antibiotic that links together rifampin and the quinolizinone ABT-719 (Fig. 10), with the aim of maintaining the potent activity of rifampin while limiting the generation of rifampin resistance [271]. Rifampin is currently the antibiotic of choice for staphylococcal prosthetic joint infections due to its potent disruption of bacterial biofilms [272]. However, rifampin is recommended to be administered alongside oral antibiotics such as LVX and CIP to minimize the occurrence of point mutations in RNA polymerase, which can cause high level resistance [273]. As such, TNP-2092 was designed to improve the therapeutic options for these persistent infections and negate the need for the administration of multiple antibiotics. TNP-2092 has been shown to retain its rifampin-mediated potency for RNA polymerase whilst simultaneously exhibiting equipotent activity against DNA gyrase and topoisomerase IV. It has a much lower propensity for resistance than its constituent molecules, as it is not a substrate for quinolone efflux. Furthermore, it is active against strains harbouring *parC* and *gyrA* mutations (responsible for quinolone resistance) and *rpoB* mutations (responsible for rifampin resistance). A TNP-2092-resistant mutant has been selected for following 26 days of serial passage. The resulting strains with MICs of >16 µg ml^−1^ and 4 µg ml^−1^ harboured five mutations: *rpoB* R484H, *gyrA* ΔL520, S84L, *parC*<R236> and H103Y [271]. TNP-2092 shows *in vitro* activity against MSSA and MRSA isolates (MIC_90_ of 0.015 µg ml^−1^) [274] and has completed a phase II double-blind, randomized, multicentre, parallel, controlled study demonstrating the safety, tolerability, appropriate pharmacokinetics and efficacy of TNP-2092 vs VAN in adults with ABSSSIs (NCT03964493). More, recently a phase I open-label study to evaluate the tissue distribution, plasma pharmacokinetics, safety and tolerability of a single 300 mg IV dose of TNP-2092 in patients undergoing total hip or knee arthroplasty was completed; however, the results have not been posted (NCT04294862).

Brilacidin (PMX30063) is a small arylamide foldamer that mimics naturally occurring AMPs (Fig. 10). It was optimized for its activity against *S. aureus* and is composed of a planar scaffold decorated with two trifluoromethane hydrophobic substitutions and four positive guanadinyl and pyridinyl substitutions [275]. It has been shown that brilacidin exposure causes a rapid depolarization of the membrane at a comparable rate to that of the clinically used lipopeptide DAP. Further transcriptomic analysis showed that, like DAP, brilacidin causes induction of the cell-wall-stress two-component systems VraRS and WalRK, a phenomenon strongly associated with lipid II-targeting antibiotics [275–277]. A serial passage resistance study showed that no significant increase in MIC occurred over a period of 16 days, demonstrating that *S. aureus* struggles to evolve resistance to brilacidin. Two phase II clinical trials have evaluated the efficacy and safety of brilacidin compared with DAP in subjects with ABSSSIs (NCT02052388 and NCT01211470). The primary endpoint of early clinical response, defined as a >20 % reduction in lesion size, was high (>90 %) in patients treated with IV brilacidin and comparable to that of the DAP-treated group. No serious AEs were documented, and all treatment-emergent AEs were mild or transient in nature, indicating an appropriate safety profile [278]. In 2015, the developer, Innovation Pharmaceuticals, announced a phase III trial further investigating brilacidin for treatment of ABSSSIs, but this has been delayed. Therapeutic use of brilacidin is being investigated in phase II trials for oral mucositis (NCT02324335), coronavirus disease 2019 (COVID-19) (NCT04784897), phase I investigation for colonic delivery (NCT04240223) and pre-clinical development for fungal infections.

Delpazolid (previously LCB01-0371) is a second-generation oxazolidinone containing an amidrazone moiety. This novel substituent to the oxazolidinone scaffold was incorporated to circumvent mitochondrial toxicity associated with prolonged LZD administration. Delpazolid subsequently proved *in vitro* to be less toxic, a feat that was mirrored in early clinical trials [279, 280]. The developer, LegoChem Biosciences, initially examined the utility of delpazolid as an anti-tuberculosis agent. An early bactericidal activity study in patients with uncomplicated tuberculosis (TB) showed promise, where delpazolid exhibited 25 % of the reduction in the log c.f.u. of the study control drug (NCT02836483) [281]. Multiple studies have shown the impressive *in vitro* and *in vivo* potency of delpazolid against *S. aureus* and enterococci [282]. The activity of delpazolid as an effective anti-MRSA agent was assessed; 100 bloodstream MRSA isolates were challenged with delpazolid and 3 comparators (VAN, DAP and LZD). The MIC_50_ and MIC_90_ for delpazolid and LZD were identical, 1 µg ml^−1^ and 2 µg ml^−1^, respectively. However, delpazolid showed a greater cumulative percentage of MICs up to 1 µg ml^−1^ than LZD (74 vs 52 %) [283]. This work directed the creation of a phase IIa study (NCT05225558) comparing the efficacy of oral delpazolid vs IV VAN in patients suffering from MRSA bacteraemia, which is currently at the recruitment stage. There has been limited investigation of the resistance potential of delpazolid in *S. aureus *. However, no significant differences in resistance rates were observed between LZD and delpazolid against XDR TB [284].

## Concluding remarks

**Fig. 11.**
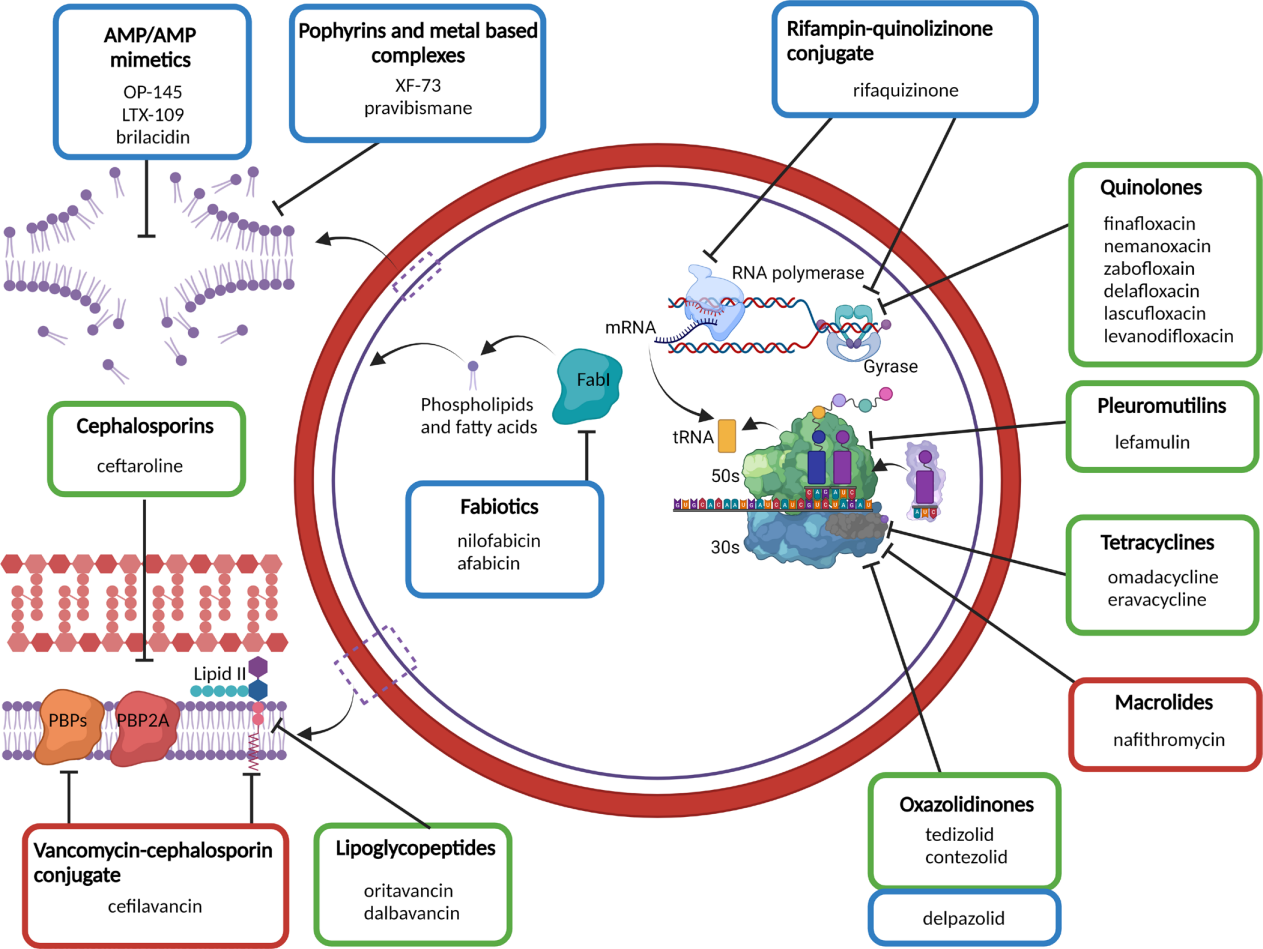
Summary of current and future anti-staphylococcal compounds**.** Colour groupings indicate recently approved antibiotics (green), antibiotics undergoing phase III trials (red) and antimicrobials undergoing phase II trials (blue)*.*

The burden of *S. aureus* antimicrobial resistance is a severe global problem. MRSA remains on the high-priority pathogen list due to the remarkable ability of this pathogen to evolve resistance to antimicrobials, disseminate widely and cause life-threatening infections. In this review, we examine the current and future pipeline of anti-staphylococcal antimicrobials (Fig. 11). Overall, there are promising signs; we discuss several novel versions of existing antibiotic classes that are active against MDR *S. aureus *. For example, significant advancements in quinolone antibiotic research have resulted in the development of drugs with enhanced stability and improved toxicity profiles, and activity against *S. aureus* strains harbouring mutations within the QRDRs and expressing efflux pumps. Second-generation oxazolidinones have shown improve potency, safety and pharmacodynamics, and a decreased propensity for resistance to emerge following prolonged *in vitro* exposure. Although successful, both examples are based on improved versions of existing classes of antibiotics and thus eventual resistance to these derivatives is more likely to emerge.

A number of the antimicrobials in phases II–III offer first-in-class compounds, but only the fabiotics afabicin and nilofabicin have a truly novel, previously unexploited target. The

diaryldiamine CRS3123 (methionyl-tRNA synthase inhibitor) and the distamycin MGB-BP-3 (DNA minor grove binder) also have novel targets but are currently under clinical investigation for *Clostridium difficile* infections (Table S1, available in the online version of this article). However, the landscape and direction of investigatory antibiotics is ever changing. Delpazolid, initially developed as an anti-TB drug, has since started phase II trials for MRSA bacteraemia. Therefore, we have also included phase II and III antibiotics that have been shown to have impressive *in vitro* activity against *S. aureus* (Table S1), and could, like delpazolid, become anti-staphylococcal antibiotics in the future. Another compound that warrants close monitoring is the FtsZ-targeting benzamide TXA709, which is currently at the beginning of its clinical journey, having recently completed phase I trials (Table S2). This is the only other anti-staphylococcal antibiotic in clinical development with a unique target.

Improvements in conjugate chemistry have ushered in a novel therapeutic approach with the development of heterodimer antibiotics with dual active targets. Cefilavancin is a covalently linked glycopeptide–cephalosporin conjugate with excellent potency against MRSA/hVISA strains. Similarly, rifaquizinone is a rifampin–quinolizinone heterodimer that simultaneously targets DNA gyrase and RNA polymerase. Along with enhanced activity, dual-acting heterodimer antibiotics may result in a lower propensity for resistance emergence during therapy. This is proving to be a popular area of antibiotic development as a third heterodimer antibiotic, DNV3837, an oxazolidinone–quinolone conjugate, is currently in phase II trials to treat *C. difficile* infections (Table S1). Future work will determine the optimal antibiotic classes to partner; current heterodimers include quinolone–macrolide, quinolone–tetracycline, quinolone–benzylpyramidine (trimethoprim) and quinolone–cephalosporin [285]. The latter combination has been used successfully to preferentially eradicate pathogenic β-lactamase-producing bacteria, over beneficial members of the microbiome [286].

Antibiotic development is an incredibly costly venture. Current estimates put the cost of developing a new antimicrobial at US $1.5 billion [287]. If we are to overcome the antibiotic resistance pandemic, small–medium-sized pharmaceutical companies that are currently the drivers of antibiotic development cannot be expected to shoulder this burden alone. This strategy will undoubtedly result in more casualties of the antibiotic development process like the hugely promising iclaprim. Fortunately, governmental healthcare systems and charitable organizations are establishing economic strategies and incentives to drive the development of novel antibiotics. These include, Combating Antibiotic-Resistant Bacteria Biopharmaceutical Accelerator (CARB-X), the Global Antibiotic Research and Development Partnership (GARDP) and the Innovative Medicines Initiative (IMI), as well as a $1 billion AMR action fund. Going forward, we need to preserve the integrity of these antibiotics. Proper antibiotic stewardship, appropriate prescribing and consumption, surveillance and increased stringency in antibiotic use in agriculture will aid in the fight to combat antibiotic resistance.

## Supplementary Data

Supplementary material 1
