# Supplementary material for: Staph wars: the antibiotic pipeline strikes back
Source: Microbiology (Reading). 2023 Sep 1;169(9):001387. doi: 10.1099/mic.0.001387 (PMC10569064; doi:10.1099/mic.0.001387)
Supplement: Supplementary material 1 [file mic-169-1387-s001.pdf]

Supplementary information

Table 1. Antibiotics under phase III and phase II clinical investigation for alternative indications that show anti-staphylococcal activity. *Antibiotics are described by company, drug class, route of administration, indication and trial number. CDAD,* Clostridium difficile-*associated diarrhoea; CDI,* C. difficile *infections; uUTI, uncomplicated urinary tract infections; IV, intra-venous; po,* Per os *(oral administration).*denotes a first in class antibiotic,**denotes a first in class antibiotic with a novel target.*

Antibiotic	Company	Drug class	Route of administration	Indication	Trial number
		**Phase III**			
Gepotidacin	GSK	Triazaacenaphthylene type II topoisomerase inhibitor*	IV, po	uUTI	NCT04010539<br>NCT04020341<br>NCT04187144
Zolidoflacin	Entasis Therapeutics	Spiropyrimidinetrione*	po	Uncomplicated gonococcal infections	NCT03959527
		**Phase II**			
MGB-BP-3	MGB Biopharma	Distamycin**	po	CDAD	NCT03824795
DNV3837	DEINOVE	Oxazolidinone-quinolone hybrid	IV	CDI	NCT03988855
CRS3123	Crestone Inc.	Diaryldiamine**	po	CDI	NCT04781387

Table 2. Antibiotics in phase I clinical trials. *Antibiotics are described by company, drug class, route of administration, indication/s and trial number. Previous developmental names are indicated in brackets. ABSSSI, acute bacterial skin and skin structure infections; BSI, bloodstream infections; CABP, community acquired bacterial pneumonia; IV, intra-venous; po,* Per os *(oral administration); PJI, prosthetic joint infections. *denotes a first in class antibiotic **denotes a first in class antibiotic with a novel target.*

Antibiotic	Company	Drug class	Route of administration	Indication	Trial number
PLG0206	Peptilogics	Antimicrobial peptide	IV	PJI	NCT05137314
Wap-8294A2 (Lotilibcin)	aRigen	Cyclic depsipeptide*	IV, topical	ABSSSI, acne vulgaris	
TXA709	TAXIS Pharmaceuticals	FtsZ benzamide**	po	MRSA infections	
DSTA4637S	Genentech	mAb rifamycin conjugate*	IV	BSI	NCT02596399<br>NCT03162250
Zifanocycline (KBP-7072)	KBP BioSciences	Tetracycline	po	CABP	NCT02454361<br>NCT04532957
TP-271	Tetraphase	Tetracycline	po	CABP	NCT03234738
BCM-0184	biocidium pharmaceuticals	Undisclosed	po, topical	MRSA infections	
RECCE327	Recce pharmaceuticals	Undisclosed	IV, topical	MRSA burn wound, BSI	
